# Treatment Patterns Across Lines of Therapy for Advanced Non‐Small Cell Lung Cancer in the United States

**DOI:** 10.1002/cam4.71736

**Published:** 2026-04-20

**Authors:** Do H. Lee, Yimeng Li, Szu‐Chun Yang, Anne C. Chiang, Pamela R. Soulos, Cary P. Gross, Shi‐Yi Wang

**Affiliations:** ^1^ Cancer Outcomes Cancer Outcomes, Public Policy, and Effectiveness Research (COPPER) Center, Yale School of Medicine New Haven Connecticut USA; ^2^ Department of Chronic Disease Epidemiology Yale School of Public Health New Haven Connecticut USA; ^3^ Department of Internal Medicine National Cheng Kung University Hospital, College of Medicine, National Cheng Kung University Tainan Tainan City Taiwan; ^4^ Department of Internal Medicine Yale School of Medicine New Haven Connecticut USA

**Keywords:** ALK, EGFR, lung cancer, PD‐L1, precision medicine, treatment pattern

## Abstract

**Introduction:**

Treatment strategies for advanced non‐small cell lung cancer (aNSCLC) have evolved, but little is known about real‐world approaches by biomarker groups across different lines of therapy (LOTs), particularly regarding discontinuation rates as a measure of effectiveness and tolerability.

**Methods:**

Using deidentified patient data from the Flatiron Health electronic health record‐derived database, we constructed a retrospective cohort study of aNSCLC patients diagnosed between January 2016 and June 2020, with follow‐up through August 2023. Patients had either anaplastic lymphoma kinase (*ALK*) rearrangements or epidermal growth factor receptor (*EGFR*) mutations or had programmed cell death‐ligand 1 (PD–L1) biomarker results (PD–L1 < 1%, 1%–49%, or ≥ 50%). For each biomarker group, we summarized treatments received across up to the sixth LOT, calculating the overall discontinuation rate and the rate due to death.

**Results:**

There were 13,369 patients in our sample (427 patients with *ALK* rearrangements, 2283 with *EGFR* mutations, 3663 PD–L1 < 1%, 3281 PD‐L1 1%–49%, and 3715 PD–L1 ≥ 50%). Treatment patterns varied across biomarker groups. The overall discontinuation rate after first‐line therapy was 31.9% for *ALK*‐rearrangement, 39.5% for *EGFR*‐mutation, and ranged from 50.6% to 57.5% for the PD–L1 groups. The discontinuation rate due to death after first‐line therapy was 8% (*ALK*‐rearrangement), 14.8% (*EGFR*‐mutation), and approximately 21% for the three PD–L1 groups. Patients with gene alterations had lower overall discontinuation rates across second through fifth LOTs than those without.

**Conclusions:**

Patients with driver alterations had lower overall discontinuation rates in subsequent therapy lines, highlighting the need for effective, tolerable treatments for those without driver alterations. Notably, a substantial proportion of patients with targetable driver alterations did not receive the corresponding TKI, underscoring real‐world gaps in biomarker‐directed treatment implementation.

## 1 Introduction

Within the United States, non‐small cell lung cancer (NSCLC) continues to be the leading cause of cancer deaths [1]. Specifically, before the last decade, five–year survival for patients with advanced NSCLC (aNSCLC) was less than 5% [2]. Up to the early 2000s, the only therapeutic options for patients who were not eligible for curative intent treatment were conventional chemotherapy and platinum‐based treatments, which demonstrated limited efficacy and low survival rates [3]. Major advancements in biomarker testing and biomarker‐specific therapies have enhanced treatment options, thereby improving health outcomes for patients with aNSCLC [4, 5, 6]. Anaplastic lymphoma kinase (*ALK*) rearrangement and epidermal growth factor receptor (*EGFR*) gene mutations are the most predominant prognostic biomarkers for NSCLC [4]. Targeted therapies, such as *ALK*‐tyrosine kinase inhibitors (TKIs) [7, 8, 9, 10, 11, 12] and *EGFR*‐TKIs [7, 13, 14, 15, 16, 17] have improved survival substantially, with estimates of five–year survival of 28% [18].

Additionally, even for patients without targetable mutations, studies suggest that programmed cell death‐ligand 1 (PD–L1) positive patients who are treated with immunotherapy have a higher response rate, longer progression‐free survival and better overall survival compared to PD–L1 negative patients [19]. Other studies have further demonstrated a potential dose‐dependent association between the intensity of PD–L1 staining and tumor responses [20].

Several studies have examined real‐world treatment patterns, [21, 22, 23] however, little is known about the patterns and subsequent lines of therapy (LOTs) by different biomarker groups. More importantly, while these new medications are generally well tolerated, patients may discontinue treatment due to a variety of reasons, such as impairment of general conditions, medication toxicity, death or disease progression [24]. Indeed, oncologists now may maintain patients on treatment beyond progression [25] and researchers have considered evaluating treatment discontinuation in pragmatic randomized trials for continuously administered therapies [26]. Examining overall discontinuation rates and the discontinuation rates due to death could contribute to a more comprehensive understanding of treatment strategies, including subsequent LOTs across biomarker groups. To address this knowledge gap, we examined the real‐world treatment patterns in aNSCLC patients with and without driver alterations (including *ALK* and *EGFR*). In patients who did not have driver alterations, we looked at PD–L1 status, including PD–L1 < 1%, PD–L1 1%–49%, and PD–L1 ≥ 50% from the United States nationwide Flatiron Health data source.

## 2 Methods

### 2.1 Data and Study Design

We conducted a retrospective cohort study with patient data extracted from the Flatiron Health electronic health record‐derived, deidentified database, comprising patient‐level data originated from ~280 US cancer clinics (~800 sites of care; primarily community oncology settings) and curated via technology‐enabled abstraction [27, 28]. Deidentified data from the Flatiron Health Database were stored in a Health Insurance Portability and Accountability Act–compliant and secure format. The Yale Human Investigation Committee determined that this study did not directly involve human participants. The study followed the Strengthening the Reporting of Observational Studies in Epidemiology (STROBE) reporting guideline [29].

### 2.2 Study Sample

We selected patients who were diagnosed with aNSCLC between January 2016 through June 2020 (Figure S1). Cases of aNSCLC were defined as patients with stage IIIB, IIIC, or IV disease according to the UICC/AJCC staging system. Staging was based on the AJCC 7th edition for cases diagnosed before July 1, 2018, and on the AJCC 8th edition for cases diagnosed on or after that date [30]. The start of our study period followed the U.S. Food and Drug Administration's (FDA) first approval of immunotherapy for aNSCLC patients in March 2015, a milestone that marked a shift in treatment trends and opened new avenues for patient care [31]. We limited our sample to aNSCLC patients who received their first LOT within 90 days after advanced diagnosis and between July 2016 and June 2020. Lines of therapy were derived using Flatiron Health's standardized algorithm, which groups drugs initiated within 28 days into a single regimen. In the first‐line setting, select agents–such as pembrolizumab, pemetrexed, and bevacizumab–may be classified as maintenance therapy when continued after other drugs are discontinued; this does not advance the line number. Additionally, restricting first‐line therapy initiation to within 90 days ensures temporal consistency, reduces immortal time bias, and aligns with clinical practice to minimize confounding from delayed treatment. Patients were followed through the last visit, death, or August 2023, whichever occurred first. Then, we selected patients with *ALK*‐rearrangements and *EGFR*‐mutations and reported PD‐L1 expression levels for those without *ALK* or *EGFR* rearrangement/mutations. Additionally, we verified that patients with PD‐L1 expression levels did not have other positive biomarker results for mesenchymal‐epithelial transition (*MET*), neurotrophic tyrosine receptor kinase (*NTRK*), rearranged during transfection (*RET*), or v‐raf murine sarcoma viral oncogene homolog B (*BRAF*). Patients with PD‐L1 expression levels were grouped into three categories based on percent staining: < 1%, 1%–49%, and ≥ 50%.

### 2.3 Statistical Analysis

All LOTs received by patients in the sample were categorized into treatment line groups based on their mechanisms of action, which refer to how the treatments function at the biological or molecular level. The categorization incorporated input from clinicians, considering factors such as therapeutic targets, patient responses, and the clinical context, to ensure a comprehensive approach that goes beyond strictly following NCCN‐recommended regimens (Table S1). For each biomarker group, we tracked the number and percentage of patients who advanced to the next LOT. We examined the distribution of treatment regimens received in the first line from 2016 to 2020 across biomarker groups. Subsequently, we calculated the distribution of treatment regimens across the second through sixth LOTs from 2016 to 2023. Although patients in the cohort received as many as ten LOTs, we only presented information up to the sixth line as the number of patients receiving more than six lines was small.

Additionally, we calculated discontinuation rates by biomarker group across LOTs. Overall discontinuation rates were calculated as the percentage of patients who did not advance from one line of therapy to the next, including discontinuations due to death. For example, first‐line treatment discontinuation rates were calculated as the proportion of patients who actively discontinued first‐line therapy without initiating second‐line therapy (numerator) among all patients who initiated first‐line therapy (denominator), including those who remained on therapy at the end of follow‐up. The numerator included patients who discontinued due to death, disease progression, or adverse effects that prevented advancement to the next line, whereas the denominator included all patients who initiated first‐line therapy, regardless of treatment status at the end of follow‐up. Discontinuation due to death was defined as the end of a line of therapy followed by patient death within 30 days of the therapy end date. Because death dates were recorded only at the month and year level, a 30–day window was applied to account for uncertainty in the exact timing of death and to capture patients who died shortly after discontinuing therapy. Patients who discontinued therapy without a documented reason, did not initiate a subsequent line of therapy, and whose death occurred beyond the 30–day window or was not observed were classified as discontinuations for unknown reasons. Specific reasons for discontinuation while alive (e.g., progression, toxicity, patient choice, lack of alternatives) were not consistently available; thus, no inference regarding cause was made. As an additional sensitivity analysis, we conducted a multivariable logistic regression to examine the association between biomarker group (ALK, EGFR, PD‐L1 < 1%, PD‐L1 1%–49%, PD‐L1 ≥ 50%) and first‐line therapy overall discontinuation within one year, defined as death within one year without receiving second‐line therapy, with all other patients considered non‐discontinued. The model was adjusted for age, race/ethnicity, sex, and ECOG performance status.

Finally, we created five sunburst charts to visually summarize the treatment regimens for patients who received subsequent LOTs, categorized by biomarker group. To demonstrate the impact of death on patients who did not receive subsequent LOTs, we also tracked patients who were unable to receive each subsequent treatment due to death. For certain outcomes that are purely descriptive in nature, formal statistical comparisons were not applicable; only summary statistics are presented. All analyses were performed using R, version 4.3.1 (R Project for Statistical Computing).

## 3 Results

Of the 13,369 patients included in our sample, 2710 had driver alterations, with 427 having *ALK* rearrangements and 2283 having *EGFR* mutations. In the 10,659 without driver alterations, 3663 had PD‐L1 < 1%, 3281 had PD‐L1 1%–49%, and 3715 had PD‐L1 ≥ 50%. Patients differed by biomarker status, especially between those with and without driver alterations (Table 1). Patients with *ALK* rearrangement were younger compared to those in the other biomarker groups: the median age at advanced diagnosis was 63, 69, 69, 70, and 70 years for *ALK*, *EGFR*, PD‐L1 < 1%, PD‐L1 1%–49% and PD‐L1 ≥ 50%, respectively. The proportion of male patients was lowest in the *EGFR* group (35.3%) compared to all other biomarker groups (*ALK*: 45.9%; PD‐L1 < 1%, 57.2%; PD‐L1 1%–49%, 55.6%; and PD‐L1 ≥ 50%, 51.7%). Patients with *ALK* or *EGFR* gene alterations had better ECOG performance status, with 47.3% and 39% having an ECOG score of 0–1, respectively, compared to between 31.2%–33.3% of patients without driver alterations. Table 2 also includes the median survival for each biomarker‐defined group and the median [IQR] duration of each line of therapy. The sunburst charts, shown in Figures 2a,b and 3a–c, capture treatment regimens for patients undergoing subsequent LOTs, while also accounting for those who were unable to receive each subsequent treatment because of death.

**TABLE 1:** Demographic characteristics of study sample (*N* = 13,369).

	Driver alterations (*N* = 2710)		No driver alteration (*N* = 10,659)		
	ALK	EGFR	PD‐L1 < 1%	PD‐L1 1%–49%	PD‐L1 ≥ 50%
	(*N* = 427)	(*N* = 2283)	(*N* = 3663)	(*N* = 3281)	(*N* = 3715)
Age, years^a^					
Median [IQR]	63 (20)	69 (16)	69 (14)	70 (14)	70 (15)
*N* (%)					
Race/ethnicity					
White	249 (58.3)	1186 (51.9)	2218 (60.6)	1978 (60.3)	2204 (59.3)
Asian	22 (5.2)	229 (10)	51 (1.4)	45 (1.4)	63 (1.7)
Black/African American	22 (5.2)	167 (7.3)	330 (9)	277 (8.4)	302 (8.1)
Other	40 (9.4)	217 (9.5)	329 (9)	298 (9.1)	291 (7.8)
Unknown	94 (22)	484 (21.2)	735 (20.1)	683 (20.8)	855 (23)
Sex: Male	196 (45.9)	805 (35.3)	2095 (57.2)	1823 (55.6)	1922 (51.7)
ECOG status					
0–1	202 (47.3)	891 (39)	1144 (31.2)	1083 (33)	1236 (33.3)
2–4	159 (37.2)	1063 (46.6)	2025 (55.3)	1760 (53.6)	1977 (53.2)
Missing	66 (15.5)	329 (14.4)	494 (13.5)	438 (13.3)	502 (13.5)

^a^ Patients with a birth year of (2020–85) or earlier may have an adjusted Birth Year in Flatiron Health datasets due to patient deidentification requirements.

**TABLE 2:** Treatment patterns and clinical outcomes: Therapy lines, duration, discontinuation, and overall survival (*N* = 13,369).

	Driver alterations		No driver alteration		
	ALK *N* = 427	EGFR *N* = 2283	PD‐L1 < 1% *N* = 3663	PD‐L1 1%–49% *N* = 3281	PD‐L1 ≥ 50% *N* = 3715
Maximum lines of therapy, *N* (%)					
One	146 (34.2)	931 (40.8)	1857 (50.7)	1724 (52.5)	2144 (57.7)
Two	134 (31.4)	635 (27.8)	978 (26.7)	879 (26.8)	931 (25.1)
Three	76 (17.8)	347 (15.2)	476 (13.0)	388 (11.8)	377 (10.1)
Four	39 (9.1)	206 (9.0)	209 (5.7)	170 (5.2)	171 (4.6)
Five	20 (4.7)	94 (4.1)	83 (2.3)	80 (2.4)	56 (1.5)
Six or More	12 (2.8)	70 (3.1)	60 (1.6)	40 (1.2)	36 (1.0)
Duration of each line of therapy (months), Median [IQR]					
First	7.8 (22.5)	8.2 (16.5)	3.1 (3.0)	3.1 (3.2)	3.6 (7.7)
Second	9.0 (17.7)	6.3 (12.4)	3.7 (6.2)	4.4 (7.3)	5.3 (11.1)
Third	5.7 (12.0)	4.3 (8.0)	3.6 (5.7)	3.7 (6.3)	4.2 (6.9)
Fourth	3.5 (8.9)	3.8 (5.3)	3.1 (4.2)	3.6 (5.7)	3.4 (4.9)
Fifth	2.7 (4.6)	3.6 (4.1)	3.2 (5.4)	3.0 (3.9)	3.3 (6.1)
Line‐specific population at risk of treatment discontinuation^a^, *N* (%)					
First	427 (100)	2283 (100)	3663 (100)	3281 (100)	3715 (100)
Second	281 (65.8)	1352 (59.2)	1806 (49.3)	1557 (47.5)	1571 (42.3)
Third	147 (34.4)	717 (31.4)	828 (22.6)	678 (20.7)	640 (17.2)
Fourth	71 (16.6)	370 (16.2)	352 (9.6)	290 (8.8)	263 (7.1)
Fifth	32 (7.5)	164 (7.2)	143 (3.9)	120 (3.7)	92 (2.5)
Overall discontinuation rates^b^, *N* (%)					
First	136 (31.9)	902 (39.5)	1854 (50.6)	1718 (52.4)	2137 (57.5)
Second	127 (45.2)	605 (44.7)	974 (53.9)	873 (56.1)	921 (58.6)
Third	67 (45.6)	337 (47)	472 (57)	385 (56.8)	369 (57.7)
Fourth	36 (50.7)	198 (53.5)	207 (58.8)	168 (57.9)	168 (63.9)
Fifth	20 (62.5)	92 (56.1)	83 (58)	78 (65)	55 (59.8)
Discontinuation rates due to death^c^, *N* (%)					
First	34 (8)	337 (14.8)	740 (20.2)	633 (19.3)	860 (23.1)
Second	41 (14.6)	262 (19.4)	512 (28.3)	434 (27.9)	406 (25.8)
Third	25 (17)	158 (22)	229 (27.7)	169 (24.9)	172 (26.9)
Fourth	20 (28.2)	104 (28.1)	100 (28.4)	93 (32.1)	85 (32.3)
Fifth	12 (37.5)	42 (25.6)	40 (28)	36 (30)	31 (33.7)
Overall survival (95% confidence interval), months					
	46.6 (37.4, 59.6)	27.1 (25.6, 28.9)	11.2 (10.7, 11.8)	12.3 (11.6, 13.0)	15.2 (14.5, 16.3)

^a^ The line‐specific population at risk of treatment discontinuation includes all patients who initiated a given line of therapy, including those still on treatment at the end of follow‐up.

^b^ Overall discontinuation rates were defined as discontinuation of a line of therapy for any reason, including death. Overall discontinuation rates are calculated as the percentage of patients who discontinue a line of therapy without advancing to the next, among all patients who initiated that therapy line, including those still on treatment at the end of follow‐up.

^c^ Discontinuation due to death includes patients who died within 30 days of ending a therapy line, capturing deaths shortly after treatment stop.

### 3.1 Treatment Pattern in First LOT

#### 3.1.1 Patients With Driver Alterations

**FIGURE 1:**
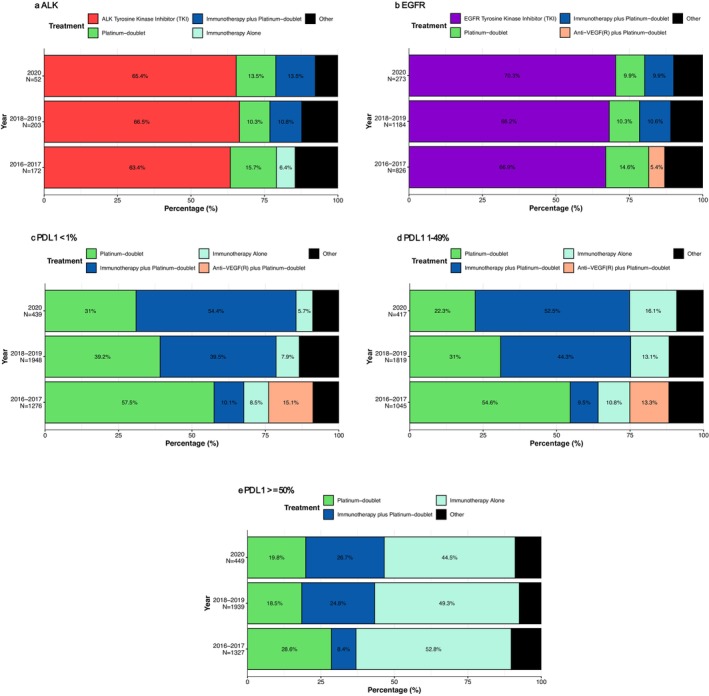
(a–e). First‐Line Treatment Patterns for Patients with aNSCLC According to Biomarker Status. All subfigures display treatments that exhibit a prevalence of ≥ 5% within each biomarker across the years as grouped in the bar charts. Otherwise, treatments demonstrating < 5% are regrouped into a category called “Other.” Year represents the year when patients received first‐line treatment.

For the 427 patients with *ALK*‐rearrangement, the most prevalent first LOT across all years from 2016 to 2020 was *ALK*‐TKI (2016–2017, 63.4%; 2018–2019 66.5%; 2020, 65.4%; Figure 1a). In 2016–2017, the second most common regimen was platinum‐doublet (15.7%), while this changed to immunotherapy plus platinum‐doublet for 2018 and later (2018–2019, 10.8%; 2020, 13.5%).

For the 2283 patients with *EGFR* mutations, the most prevalent first‐line therapy across all years was *EGFR*‐TKI (Figure 1b; 2016–2017, 66.9%; 2018–2019, 68.2%; 2020, 70.3%). Platinum‐doublet was the second most prevalent regimen in 2016–2017 (14.6%), whereas immunotherapy plus platinum‐doublet became the second most prevalent treatment in 2018–2019 (10.6%) and 2020 (9.9%). Anti‐VEGF(R) plus platinum‐doublet was the next most prevalent treatment in 2016–2017 (5.4%). However, its prevalence decreased to less than 5% during the subsequent years.

#### 3.1.2 Patients Without Driver Alterations

For the 3663 patients in the PD‐L1 < 1% group, platinum‐doublet was the most prevalent first‐line treatment from 2016 to 2017 but started to diminish in prominence across the years (Figure 1c; 2016–2017, 57.5% (95% CI of 54.8–60.2); 2018–2019, 39.2% (95% CI of 37–41.4); 2020, 31% (95% CI of 26.8–35.5)). The decreasing trend over the three time periods was statistically significant (Cochran–Armitage trend test, *p* < 0.001), indicating that the observed decline is unlikely due to random variation. The variation most likely occurs because guidelines recommended platinum doublet, with or without anti‐angiogenesis inhibitors, before the approval of the KEYNOTE‐189 platinum doublet immunotherapy combination.^31^ In alignment with the approval of KEYNOTE‐024 immunotherapy plus chemotherapy, immunotherapy plus platinum‐doublet replaced platinum‐doublet in prominence (2016–2017, 10.1%; 2018–2019, 39.5%; 2020, 54.4%). Moreover, anti‐VEGF(R) plus platinum‐doublet demonstrated a prevalence of 15.1% between 2016 and 2017, but the use diminished to less than 5% after 2018.

The 3281 patients in the PD‐L1 1%–49% group demonstrated similar patterns of care compared to the PD‐L1 < 1% patients: platinum‐doublet use decreased across the years (Figure 1d; 2016–2017, 54.6% (95% CI of 51.6–57.6); 2018–2019, 31% (95% CI of 28.9–33.1); 2020: 22.3% (95% CI of 18.6–26.5); Cochran–Armitage trend test, *p* < 0.001). The prevalence of immunotherapy alone rose across the years (2016–2017:10.8% (95% CI of 9.1–12.8); 2018–2019:13.1% (95% CI of 11.6–14.7); 2020:16.1% (95% CI of 12.9–19.9); Cochran Armitage trend test, *p* = 0.005). Parallel to PD‐L1 < 1% treatment patterns, anti‐VEGF(R) plus platinum‐doublet demonstrated a prominence of 13.3% in 2016–2017 but declined to less than 5% among treatments across the remaining years.

The 3715 patients in the PD‐L1 ≥ 50% group demonstrated treatment patterns distinct from both the PDL < 1% and 1%–49% patients. Immunotherapy alone was the most prevalent treatment across the years (Figure 1e; 2016–2017, 52.8%; 2018–2019, 49.3%; 2020, 44.5%). Immunotherapy plus platinum‐doublet started with lower prevalence in earlier years and progressed to be the second most prominent treatment from 2018 onward (2016–2017, 8.4%; 2018–2019, 24.8%; 2020, 26.7%). Platinum‐doublet started with high prevalence in 2016–2017 but maintained a diminished prominence afterwards (2016–2017, 28.6% (95% CI of 26.2–31.1); 2018–2019, 18.5% (95% CI of 16.8–20.3); 2020, 19.8% (95% CI of 16.4–23.8); Cochran Armitage trend test, *p* < 0.001).

As expected, immunotherapy monotherapy was used less frequently in the PD‐L1 < 1% cohort, especially in earlier years when it was not guideline‐recommended. In our cohort, 7.8% of patients with PD‐L1 < 1% received first‐line immunotherapy alone, compared to 12.7% of those with PD‐L1 1%–49% (Figure 3a–c). Among the 287 PD‐L1 < 1% patients receiving monotherapy, most (179 patients, 62.4%) initiated treatment after 2018, following guideline updates.

### 3.2 Treatment Variation in Second and Third LOTs

#### 3.2.1 Patients With Driver Alterations

For the *ALK*‐positive patients, the most prevalent second‐line therapy was *ALK*‐TKI across all years (Figure S2a.1; 2016–2017, 69.1%; 2018–2019, 69.8%; 2020, 81.2%). Platinum doublet was used in at least 5% of patients during 2016 through 2019, but not in subsequent years. Among patients who received third‐line therapy, *ALK*‐TKI remained prominent across all years (Figure S2a.2). However, there was marked variation in the other treatment regimens received across the years.

For *EGFR*‐positive patients, *EGFR*‐TKI was commonly used as a second‐line therapy across all years but decreased over time (Figure S2b.1; 2016–2017, 61.9% (95% CI of 57.8–65.8); 2018–2019, 45.1% (95% CI of 41.3–49); 2020, 32.9% (95% CI of 25.6–41); Cochran Armitage trend test, *p* < 0.001). Resembling third‐line treatment patterns observed in *ALK*‐positive patients, *EGFR*‐positive patients demonstrated greater variety in treatments with at least 5% prevalence across all years in the third LOT compared to the second (Figure S2b.2).

#### 3.2.2 Patients Without Driver Alterations

For the three groups of PD‐L1, immunotherapy alone maintained higher distributions throughout the years for second‐line therapy (Figure S3a.1–c.1). In third‐line therapy, chemotherapy without platinum became more popular than immunotherapy alone across all three groups of PD‐L1 across the years (Figure S3a.2–c.2).

#### 3.2.3 Overall Discontinuation Rates Across All Lines of Therapy

Within a 3‐year follow‐up period, patients with *ALK*‐rearranged and *EGFR*‐mutated aNSCLC were more likely to receive three or more LOTs than those of patients in the three PD‐L1 groups (Table 2). Additionally, overall discontinuation rates from first through fifth LOT in patients with driver alterations (*ALK* and *EGFR*) were lower than those of patients without, indicating patients with driver alterations were able to continue treatment within each LOT. In contrast, patients in the three PD‐L1 groups had higher overall discontinuation rates; in addition, within each LOT, more than 19% of patients discontinued their treatment in the presence of death. For instance, within the 3–year follow‐up period, those in the no driver alteration cohorts had higher proportions of patients who experienced death (78.3% vs. 66.5%; Chi‐square test, *p* < 0.001). Similarly, the multivariable logistic regression model for treatment overall discontinuation at one year post‐first‐line therapy showed significant differences across biomarker groups (*p* < 0.001, Table S2). With EGFR as the reference, the odds ratio and 95% confidence interval for PD‐L1 < 1% was 2.69 (2.39, 3.03); for PD‐L1 1%–49% was 2.37 (2.11, 2.68); and for PD‐L1 ≥ 50% was 2.01 (1.79, 2.26).

#### 3.2.4 Patients With Driver Alterations

**FIGURE 2:**
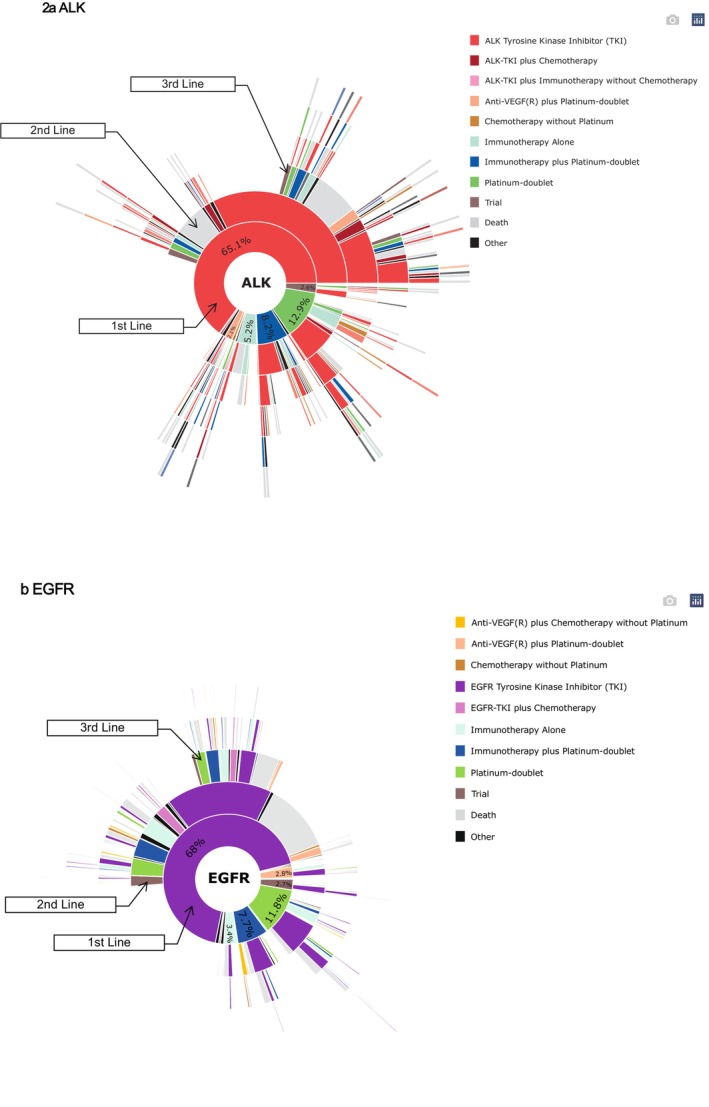
(a, b). Sunburst Plots Showing Treatment Patterns for aNSCLC with Driver Alterations. All subfigures display treatments that exhibit a prevalence of ≥ 5% within each biomarker across the years as grouped in the bar charts. Otherwise, treatments demonstrating < 5% are regrouped into a category called “Other.” Sunburst plots display observed treatment transitions or death. Patients who discontinued therapy for unknown reasons or who remained on therapy at last follow‐up were classified as having no observed transition and are shown as blank white space.

For patients with *ALK*‐rearrangements, overall discontinuation rates for the first‐ and second‐line treatments were 31.9% and 45.2%, respectively, with 8% and 14.6% of those discontinuations attributed to death. Among those who received an ALK‐TKI as their first‐line treatment (*N* = 278), 3.2% stayed on the first‐line therapy, 50% received a different ALK‐TKI as the second‐line therapy, 10.4% proceeded to a non‐ALK‐TKI second‐line therapy, and 8.6% discontinued due to death, whereas 27.7% were discontinued for unknown reasons (Figure 2a). In contrast, among those who received non‐ALK‐TKI treatment as their first‐line therapy (*N* = 149), only ≤ 3.4% stayed on the first‐line therapy, 40.3% received an ALK‐TKI as the second‐line therapy, 35.6% proceeded to a non‐ALK‐TKI second‐line therapy, and 6.7% discontinued due to death, whereas ≥ 14.1% were discontinued for unknown reasons. By the sixth line of treatment, overall discontinuation was 62.5% with 37.5% due to death.

For patients with *EGFR*‐mutated aNSCLC, overall discontinuation rates for the first‐ and second‐line treatments were 39.5% and 44.7%, respectively, with 14.8% and 19.4% discontinuation due to death. Among those who received an *EGFR*‐TKI as their first‐line treatment (*N* = 1552), 1.7% stayed on the first‐line therapy, 53% proceeded to a second‐line therapy (including 26% receiving another *EGFR*‐TKI as the second‐line therapy), and 16.8% discontinued due to death and 28.7% due to unknown reasons (Figure 2b). In contrast, among those who received non‐*EGFR*‐TKI treatment as their first‐line therapy (*N* = 731), ≤ 0.7% stayed on the first‐line therapy, 39% received an *EGFR*‐TKI as the second‐line therapy, 33.5% proceeded to a non‐*EGFR*‐TKI second‐line therapy, and 10.8% discontinued due to death. At the sixth LOT, the overall discontinuation rate was 56.1%, with 25.6% attributable to death.

#### 3.2.5 Patients Without Driver Alterations

**FIGURE 3:**
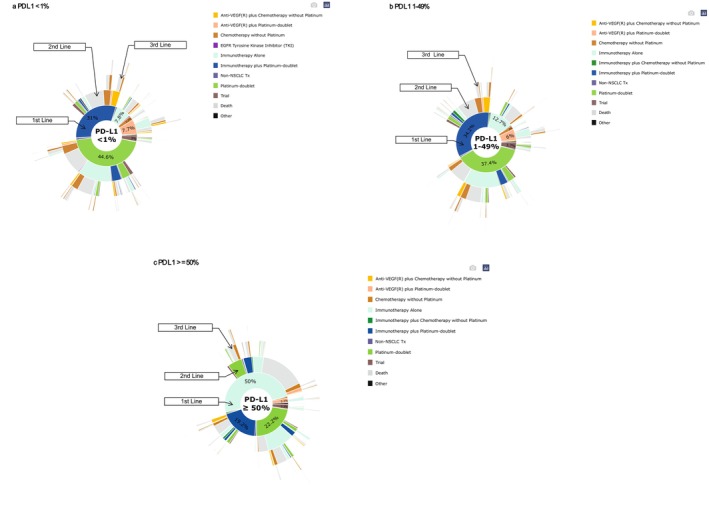
(a–c). Sunburst Plots Showing Treatment Patterns for aNSCLC with No Driver Alteration. All subfigures display treatments that exhibit a prevalence of ≥ 5% within each biomarker across the years as grouped in the bar charts. Otherwise, treatments demonstrating < 5% are regrouped into a category called “Other.” Sunburst plots display observed treatment transitions or death. Patients who discontinued therapy for unknown reasons or who remained on therapy at last follow‐up were classified as having no observed transition and are shown as blank white space.

Across all three PD‐L1 groups, overall discontinuation rates were mostly attributable to death (Figure 3a–c). For example, overall discontinuation rate for the first‐line treatment was 50.6% for PD‐L1% < 1%, 52.4% for PD‐L1 1%–49%, and 57.5% for PD‐L1% ≥ 50%, whereas discontinuation rate due to death was 20.2%, 19.3%, and 23.1%, respectively (Table 2). Among patients with PD‐L1 ≥ 50% who received immunotherapy alone as their first‐line treatment (*N* = 1856), ≤ 0.3% stayed in the first‐line therapy, 32.3% proceeded to a second‐line treatment, and 30.5% discontinued due to death and ≥ 36.9% due to unknown reasons. Yet it was 0%, 38.3%, 17.4%, and 44.2%, respectively, for PD‐L1 ≥ 50% who received immunotherapy plus platinum as their first‐line treatment (*N* = 712).

#### 3.2.6 Treatment Pattern Combinations of the First Three LOTs

The sunburst plots in Figures 2a,b and 3a–c focused on the hierarchical distribution of treatments across subsequent LOTs. Among patients with *ALK*‐rearranged aNSCLC who received three LOTs, there were 68 different treatment pattern combinations based on treatment categories received in the first, second, and third LOTs. For *EGFR*‐positive patients who received three LOTs, there were 283 different treatment combinations. For the three groups of PD‐L1, the PD‐L1 < 1% patients had the greatest treatment variation in the first three LOTs (the number of treatment combinations: 293 for PD‐L1 < 1%, 259 for PD‐L1 1%–49%, 226 for PD‐L1 ≥ 50%, respectively). Because we had already categorized treatments based on their mechanism (e.g., 5 different *ALK*‐TKIs were grouped as one category), the number of treatment pattern combinations would be even larger if we investigated the unique treatment regimens patients received.

## 4 Discussion

In this retrospective cohort study of patients diagnosed with aNSCLC, we described how treatment patterns varied by biomarker status. Given the current movement toward precision medicine, biomarker status plays a critical role in treatment decision‐making. Extensive literature demonstrates that patients with NSCLC harboring actionable driver mutations such as EGFR or ALK have better prognosis and survival outcomes due to the availability of effective targeted therapies [18, 32], whereas those without such alterations face limited options and poorer outcomes, underscoring the need for more effective and well‐tolerated treatments [33]. Our study highlights the importance of collecting biomarker data in clinical research. Furthermore, in the first LOT, *ALK*‐TKI and *EGFR*‐TKI maintained ubiquity for patients with *ALK*‐rearrangements and *EGFR*‐mutations, whereas among PD‐L1 patients, there was no dominant regimen. Our study identified the three PD‐L1 groups as instances of clinical equipoise, a variety of recommended regimens without the preferred/best one, indicating a need for future research on determining the best regimens among these populations.

Our study has several important clinical implications. First, the overall discontinuation rates for first‐line therapy differed by biomarker group, ranging from 31.9% in *ALK*‐positive patients to 57.5% in PD‐L1 ≥ 50% patients. However, direct comparison of the overall discontinuation rates by biomarker groups may be misleading. Several factors, including regimen's profiles, such as tolerability and efficacy, could contribute to different overall discontinuation rates [34]. We separated discontinuations due to death from those in patients who were still alive for other reasons. For example, the first‐line therapy discontinuation rate due to death was 8% in patients with *ALK*‐rearrangement but 23.1% in patients with PD‐L1 ≥ 50%. Nevertheless, among patients with *ALK*‐rearrangements and *EGFR*‐mutations who received their respective TKIs, the percentage of patients who died was relatively small, reiterating the benefits of TKIs and its favorable tolerability profiles.

Second, approximately one third of *ALK*‐ and *EGFR*‐positive patients did not receive the corresponding TKIs as their first‐line therapy. Of these patients, several received platinum doublets, presumably due to delays in testing prior to the initiation of first‐line therapy. A non‐trivial proportion of patients received immunotherapy plus platinum‐doublet after 2018, despite evidence demonstrating that this approach was less effective and costlier than TKIs [35]. Efforts to promote appropriate treatments among these patients would save costs and improve survival.

Third, there was a dramatic increase in use of immunotherapy plus platinum‐doublet over time among the three PD‐L1 groups, which echoed the FDA approval of pembrolizumab in the first‐line setting in 2018 [36]. Interestingly, for aNSCLC patients with PD‐L1 ≥ 50%, the use of immunotherapy alone as the first‐line therapy decreased over time, suggesting a lack of physicians' confidence in using immunotherapy alone. This warrants a direct comparison between immunotherapy alone and immunotherapy plus platinum‐doublet.

Fourth, because new generation *ALK*‐TKIs, such as lorlatinib, are available as the second‐line therapy after first‐line *ALK*‐TKIs, we observed that many *ALK*‐positive patients still received an *ALK*‐TKI as their second‐line therapy. However, upon FDA approval of osimertinib as the first‐line therapy for *EGFR*‐positive patients in 2018, there has been no alternative *EGFR*‐TKI treatment option after progression. Therefore, use of *EGFR*‐TKI as second‐line therapy decreased over time. Also, because immunotherapy agents were moved from the second‐line therapy to the first‐line therapy starting in late 2016, the proportion of patients who received immunotherapy agents as their second LOT decreased over time.

Fifth, the heterogeneity in treatment patterns beyond second‐line therapy highlights the lack of clear, evidence‐based guidelines for later‐line management of aNSCLC. Unlike first‐ and second‐line treatments, which are generally well‐defined by clinical trials and biomarker‐driven protocols, therapy selection beyond the second line is often guided by individual patient factors–such as performance status, comorbidities, prior treatment response and tolerability–as well as provider experience and evolving clinical practice. Additionally, the availability and approval timelines of newer agents during the study period may have contributed to diverse prescribing behaviors. This heterogeneity highlights the uncertainty that still exists in the management of aNSCLC in later lines and reinforces the importance of generating real‐world evidence to support clinical decision‐making where guidelines remain limited.

Our study has several strengths. A key strength of this study is the use of a large, diverse EHR‐derived dataset encompassing community and academic practices. Prior benchmarking analyses have demonstrated that Flatiron Health cohorts closely resemble national datasets such as SEER and NCDB, differing mainly by a modest under‐representation of patients aged 80 years or older, supporting the generalizability of our findings [37]. We described overall discontinuation rates and accounted for patients who did not receive subsequent LOTs due to death across different biomarkers. Moreover, our sunburst charts were able to record the sequences of treatment regimens, which Sankey diagrams cannot do. Also, our results, derived from a population‐based data, are thus generalizable to a broad and diverse patient population. However, a limitation is the lack of information in terms of adverse effects, comorbid conditions, and patient preference that may influence patients who did not receive subsequent LOTs. Another limitation is the sample size per biomarker group, which was too small to analyze yearly trends effectively. Lastly, a key limitation of this study is the potential misclassification of treatment transitions due to reliance on structured EHR algorithms. While the Flatiron algorithm includes rules to identify maintenance therapy in the first‐line setting, some clinical nuances and atypical treatment patterns may not be fully captured. Additionally, incomplete or imprecise oral therapy data and variability in documentation may affect the accuracy of line assignments.

## 5 Conclusions

In summary, biomarker status and treatment timing are crucial factors for research examining treatment variation. Consistent with guideline recommendation, patients with *ALK*‐rearrangements and *EGFR*‐mutations were likely to receive their respective TKIs. However, a non‐trivial proportion of patients did not receive the corresponding TKIs, raising quality of care concerns. Notably, patients without driver alterations were more likely to discontinue subsequent LOTs than those with them, indicating the need to provide effective and tolerable therapies for patients without driver alterations.

## Author Contributions

**Do H. Lee:** writing – original draft, writing – review and editing, methodology, conceptualization, data curation. **Yimeng Li:** writing – review and editing, methodology. **Szu‐Chun Yang:** writing – review and editing, methodology. **Anne C. Chiang:** writing – review and editing, methodology. **Pamela R. Soulos:** writing – review and editing, methodology. **Cary P. Gross:** writing – review and editing, methodology. **Shi‐Yi Wang:** writing – review and editing, conceptualization, methodology, supervision.

## Funding

This study was supported by a Research Scholar Grant, RSG‐23‐723426‐01‐HOPS, from the American Cancer Society.

## Conflicts of Interest

Cary P. Gross: Dr. Gross has received research funding from the NCCN Foundation (funds provided by Astra‐Zeneca), as well as funding from Johnson and Johnson to help devise and implement new approaches to sharing clinical trial data. The remaining authors declare no conflicts of interest.

## Supporting information


**Figure S1:** Sample Selection Criteria Flow Chart.
**Figures S2a:**,b. Second and Third‐Line Treatment Patterns for aNSCLC with Driver Alterations.
**Figures S2a:**1–b.1 depict second‐line treatment patterns, and panels 2a.2–b.2 depict third‐line treatment patterns.
All subfigures display treatments that exhibit a prevalence of ≥ 5% within each biomarker across the years as grouped in the bar charts. Otherwise, treatments demonstrating < 5% are regrouped into category called “Other.” Year represents the year when patients received first‐line treatment.
**Figure S3a:**–c. Second and Third‐Line Treatment Patterns for aNSCLC without Driver Alterations.
**Figures S3a:**1–c.1 depict second‐line treatment patterns, and panels 3a.2–c.2 depict third‐line treatment patterns.
All subfigures display treatments that exhibit a prevalence of ≥ 5% within each biomarker across the years as grouped in the bar charts. Otherwise, treatments demonstrating < 5% are regrouped into category called “Other.” Year represents the year when patients received first‐line treatment.

**Table S1:** Categorization of Regimens Based on Clinician Input, Therapeutic Targets, and Clinical Contexts Beyond NCCN Guidelines.

**Table S2:** Adjusted Association of Biomarker Group with Treatment Overall Discontinuation at One Year Post‐First Line Therapy.

## Data Availability

The data that support the findings of this study are available from publicationsdataaccess@flatiron.com . Restrictions apply to the availability of these data, which were used under license for this study. Data are available from the author(s) with the permission of PublicationsDataAccess@flatiron.com.
