# Supplementary material for: Treatment Patterns Across Lines of Therapy for Advanced Non‐Small Cell Lung Cancer in the United States
Source: Cancer Med. 2026 Apr 20;15(4):e71736. doi: 10.1002/cam4.71736 (PMC13094514; doi:10.1002/cam4.71736)
Supplement: Supplementary file 1 — Figure S1: Sample Selection Criteria Flow Chart. Figures S2a:,b. Second and Third‐Line Treatment Patterns for aNSCLC with Driver Alterations. Figures S2a:1–b.1 depict second‐line treatment patterns, and panels 2a.2–b.2 depict third‐line treatment patterns. All subfigures display treatments that exhibit a prevalence of ≥ 5% within each biomarker across the years as grouped in the bar charts. Otherwise, treatments demonstrating < 5% are regrouped into category called “Other.” Year represents the year when patients received first‐line treatment. Figure S3a:–c. Second and Third‐Line Treatment Patterns for aNSCLC without Driver Alterations. Figures S3a:1–c.1 depict second‐line treatment patterns, and panels 3a.2–c.2 depict third‐line treatment patterns. All subfigures display treatments that exhibit a prevalence of ≥ 5% within each biomarker across the years as grouped in the bar charts. Otherwise, treatments demonstrating < 5% are regrouped into category called “Other.” Year represents the year when patients received first‐line treatment. [file CAM4-15-e71736-s001.zip › cam471736-sup-0002-FigureS1-S2@Supplemental Figure 2a.1 ALK.pdf]

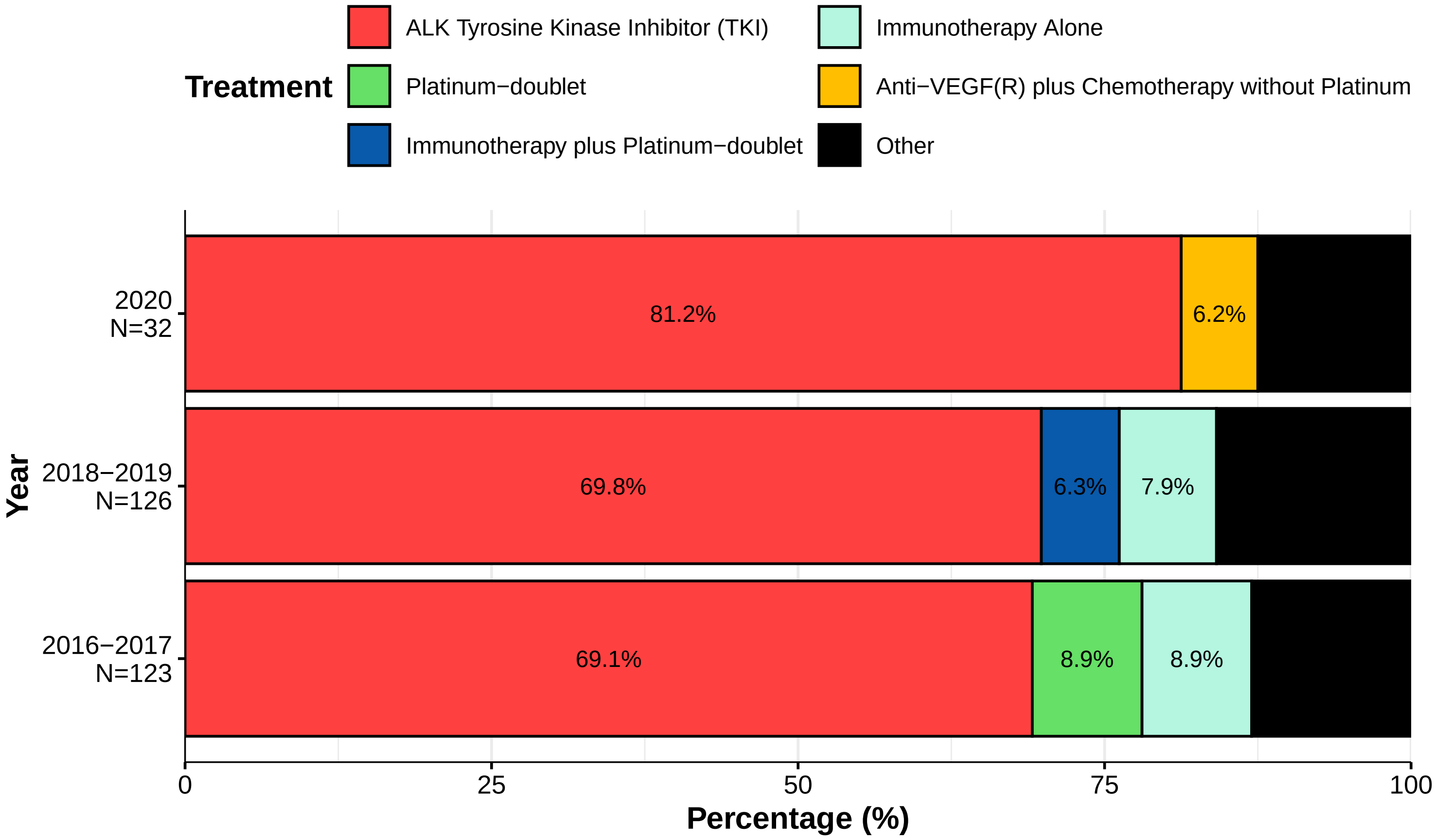

Treatment
ALK Tyrosine Kinase Inhibitor (TKI)
Platinum−doublet
Immunotherapy plus Platinum−doublet
Immunotherapy Alone
Anti−VEGF(R) plus Chemotherapy without Platinum
Other
Year
2020
N=32
2018−2019
N=126
2016−2017
N=123
81.2%
6.2%
69.8%
6.3%
7.9%
69.1%
8.9%
8.9%
0
25
50
75
100
Percentage (%)
