# Supplementary figures and images for: Treatment Patterns Across Lines of Therapy for Advanced Non‐Small Cell Lung Cancer in the United States
Source: Cancer Med. 2026 Apr 20;15(4):e71736. doi: 10.1002/cam4.71736 (PMC13094514; doi:10.1002/cam4.71736)

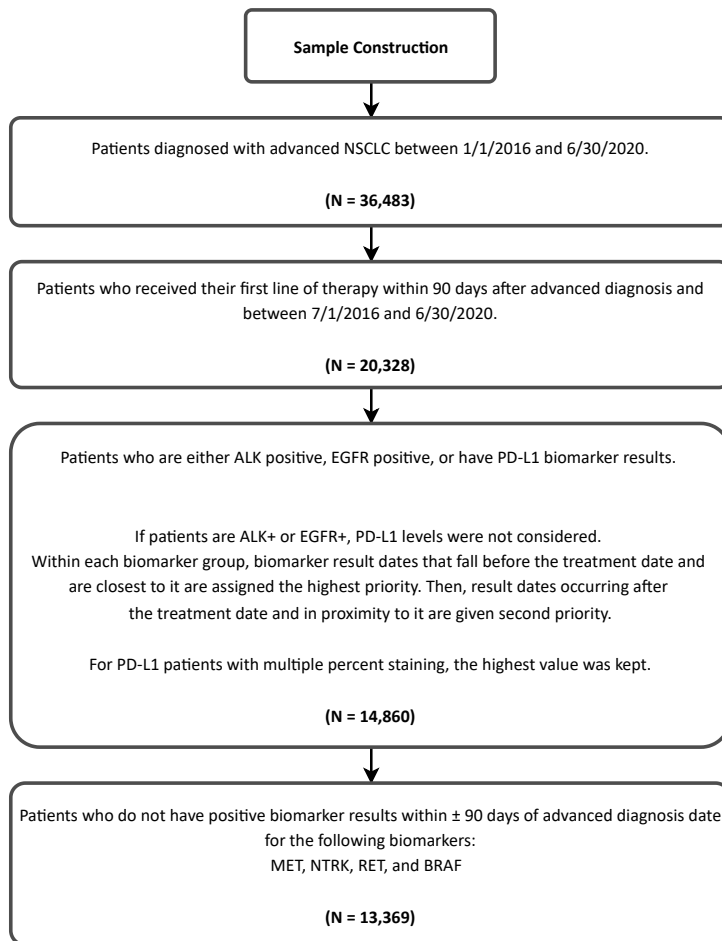

Supplement: Supplementary file 1 — Figure S1: Sample Selection Criteria Flow Chart. Figures S2a:,b. Second and Third‐Line Treatment Patterns for aNSCLC with Driver Alterations. Figures S2a:1–b.1 depict second‐line treatment patterns, and panels 2a.2–b.2 depict third‐line treatment patterns. All subfigures display treatments that exhibit a prevalence of ≥ 5% within each biomarker across the years as grouped in the bar charts. Otherwise, treatments demonstrating < 5% are regrouped into category called “Other.” Year represents the year when patients received first‐line treatment. Figure S3a:–c. Second and Third‐Line Treatment Patterns for aNSCLC without Driver Alterations. Figures S3a:1–c.1 depict second‐line treatment patterns, and panels 3a.2–c.2 depict third‐line treatment patterns. All subfigures display treatments that exhibit a prevalence of ≥ 5% within each biomarker across the years as grouped in the bar charts. Otherwise, treatments demonstrating < 5% are regrouped into category called “Other.” Year represents the year when patients received first‐line treatment. [file CAM4-15-e71736-s001.zip › cam471736-sup-0001-FigureS1@Supplemental Figure 1.pdf]

# Treatment

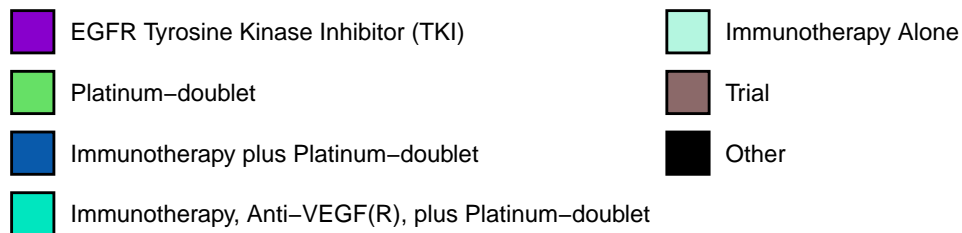

Year

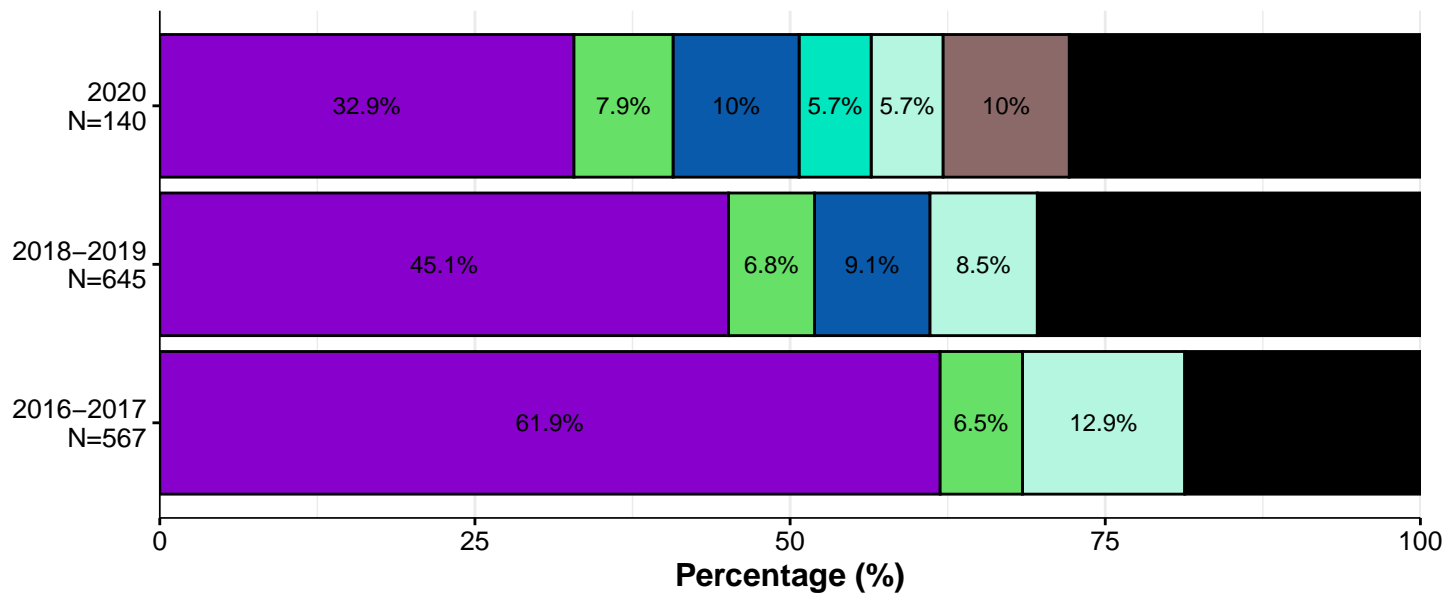

Supplement: Supplementary file 1 — Figure S1: Sample Selection Criteria Flow Chart. Figures S2a:,b. Second and Third‐Line Treatment Patterns for aNSCLC with Driver Alterations. Figures S2a:1–b.1 depict second‐line treatment patterns, and panels 2a.2–b.2 depict third‐line treatment patterns. All subfigures display treatments that exhibit a prevalence of ≥ 5% within each biomarker across the years as grouped in the bar charts. Otherwise, treatments demonstrating < 5% are regrouped into category called “Other.” Year represents the year when patients received first‐line treatment. Figure S3a:–c. Second and Third‐Line Treatment Patterns for aNSCLC without Driver Alterations. Figures S3a:1–c.1 depict second‐line treatment patterns, and panels 3a.2–c.2 depict third‐line treatment patterns. All subfigures display treatments that exhibit a prevalence of ≥ 5% within each biomarker across the years as grouped in the bar charts. Otherwise, treatments demonstrating < 5% are regrouped into category called “Other.” Year represents the year when patients received first‐line treatment. [file CAM4-15-e71736-s001.zip › cam471736-sup-0003-FigureS1-S2@Supplemental Figure 2b.1 EGFR.pdf]

# Treatment

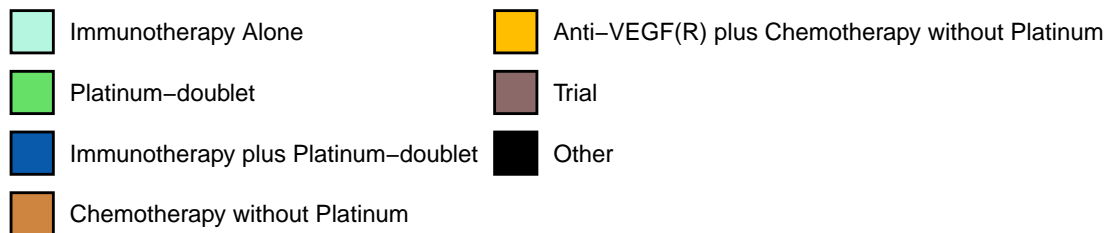

Year

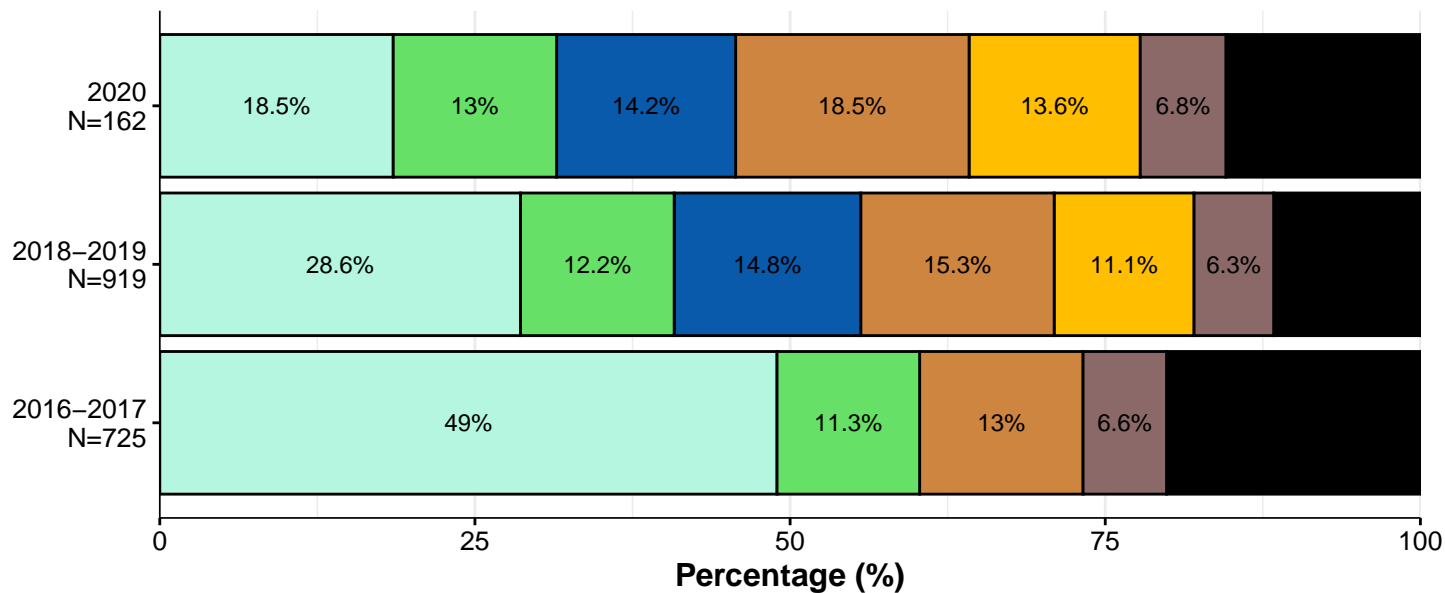

Supplement: Supplementary file 1 — Figure S1: Sample Selection Criteria Flow Chart. Figures S2a:,b. Second and Third‐Line Treatment Patterns for aNSCLC with Driver Alterations. Figures S2a:1–b.1 depict second‐line treatment patterns, and panels 2a.2–b.2 depict third‐line treatment patterns. All subfigures display treatments that exhibit a prevalence of ≥ 5% within each biomarker across the years as grouped in the bar charts. Otherwise, treatments demonstrating < 5% are regrouped into category called “Other.” Year represents the year when patients received first‐line treatment. Figure S3a:–c. Second and Third‐Line Treatment Patterns for aNSCLC without Driver Alterations. Figures S3a:1–c.1 depict second‐line treatment patterns, and panels 3a.2–c.2 depict third‐line treatment patterns. All subfigures display treatments that exhibit a prevalence of ≥ 5% within each biomarker across the years as grouped in the bar charts. Otherwise, treatments demonstrating < 5% are regrouped into category called “Other.” Year represents the year when patients received first‐line treatment. [file CAM4-15-e71736-s001.zip › cam471736-sup-0004-FigureS1-S3@Supplemental Figure 3a.1 PDL1 Less 1.pdf]

# Treatment

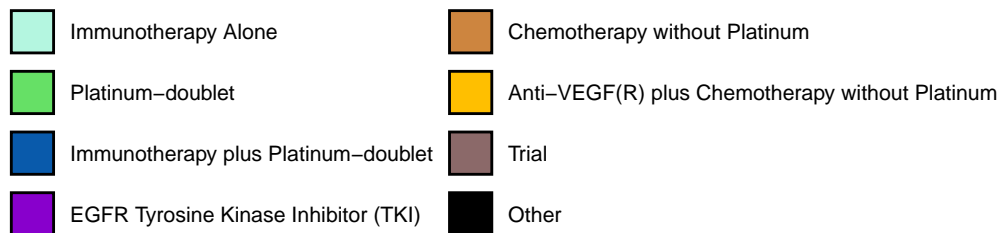

Year

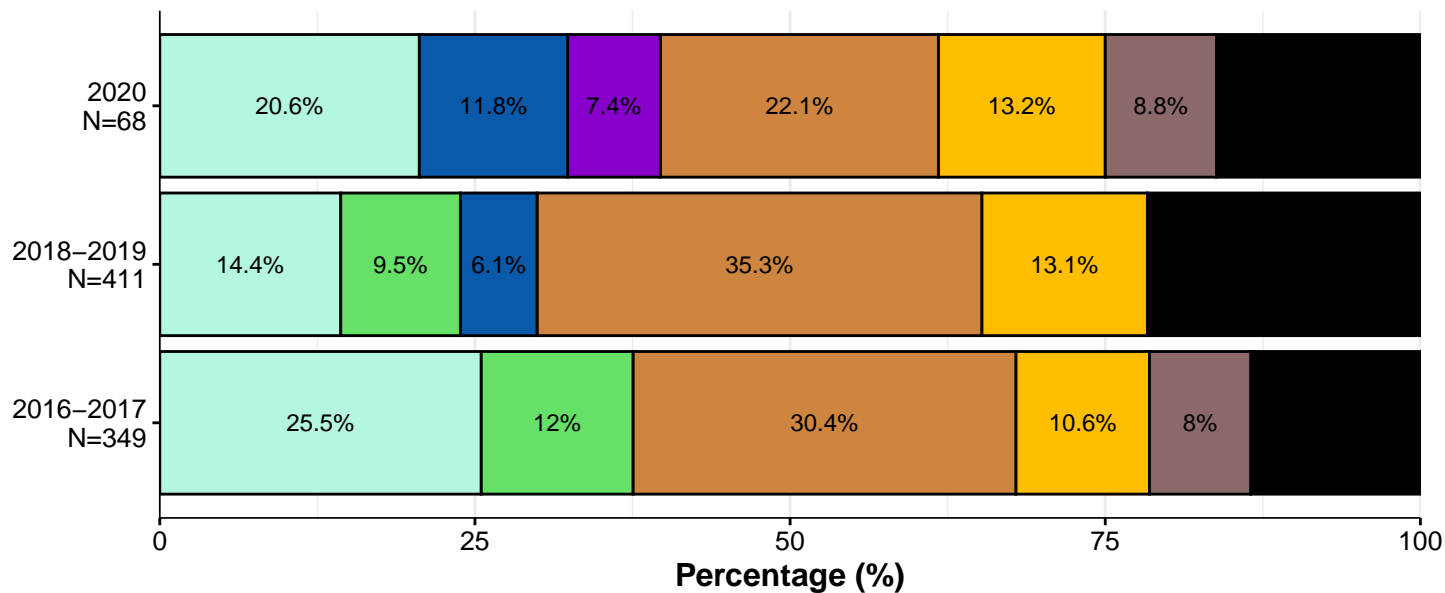

Supplement: Supplementary file 1 — Figure S1: Sample Selection Criteria Flow Chart. Figures S2a:,b. Second and Third‐Line Treatment Patterns for aNSCLC with Driver Alterations. Figures S2a:1–b.1 depict second‐line treatment patterns, and panels 2a.2–b.2 depict third‐line treatment patterns. All subfigures display treatments that exhibit a prevalence of ≥ 5% within each biomarker across the years as grouped in the bar charts. Otherwise, treatments demonstrating < 5% are regrouped into category called “Other.” Year represents the year when patients received first‐line treatment. Figure S3a:–c. Second and Third‐Line Treatment Patterns for aNSCLC without Driver Alterations. Figures S3a:1–c.1 depict second‐line treatment patterns, and panels 3a.2–c.2 depict third‐line treatment patterns. All subfigures display treatments that exhibit a prevalence of ≥ 5% within each biomarker across the years as grouped in the bar charts. Otherwise, treatments demonstrating < 5% are regrouped into category called “Other.” Year represents the year when patients received first‐line treatment. [file CAM4-15-e71736-s001.zip › cam471736-sup-0005-FigureS1-S3@Supplemental Figure 3a.2 PDL1 Less 1.pdf]

# Treatment

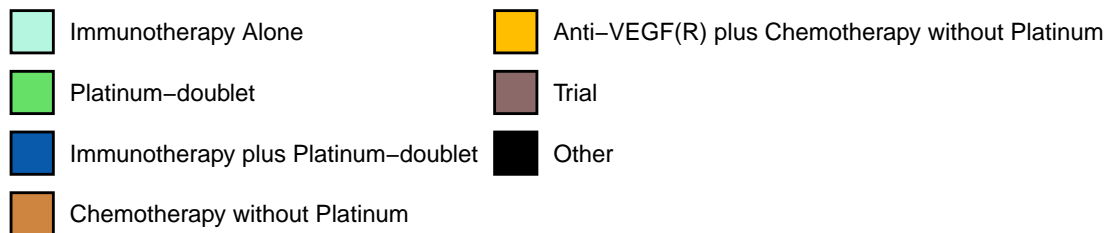

Year

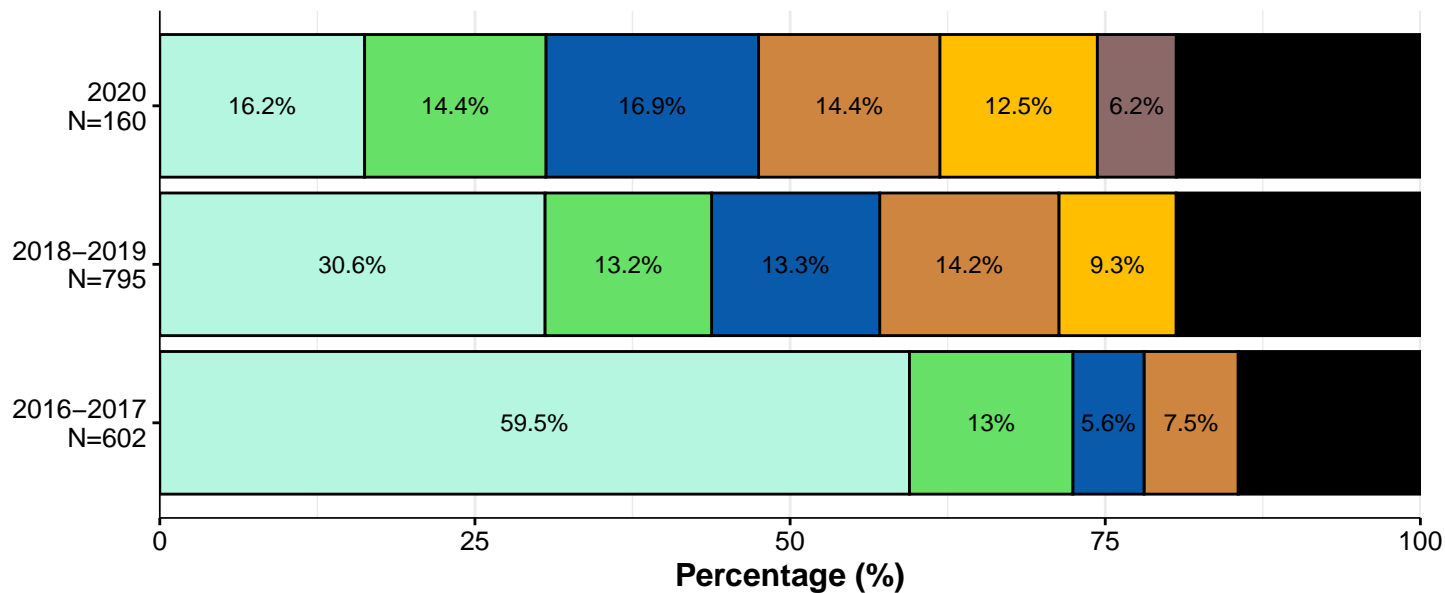

Supplement: Supplementary file 1 — Figure S1: Sample Selection Criteria Flow Chart. Figures S2a:,b. Second and Third‐Line Treatment Patterns for aNSCLC with Driver Alterations. Figures S2a:1–b.1 depict second‐line treatment patterns, and panels 2a.2–b.2 depict third‐line treatment patterns. All subfigures display treatments that exhibit a prevalence of ≥ 5% within each biomarker across the years as grouped in the bar charts. Otherwise, treatments demonstrating < 5% are regrouped into category called “Other.” Year represents the year when patients received first‐line treatment. Figure S3a:–c. Second and Third‐Line Treatment Patterns for aNSCLC without Driver Alterations. Figures S3a:1–c.1 depict second‐line treatment patterns, and panels 3a.2–c.2 depict third‐line treatment patterns. All subfigures display treatments that exhibit a prevalence of ≥ 5% within each biomarker across the years as grouped in the bar charts. Otherwise, treatments demonstrating < 5% are regrouped into category called “Other.” Year represents the year when patients received first‐line treatment. [file CAM4-15-e71736-s001.zip › cam471736-sup-0006-FigureS1-S49@Supplemental Figure 3b.1 PDL1 1to49.pdf]

# Treatment

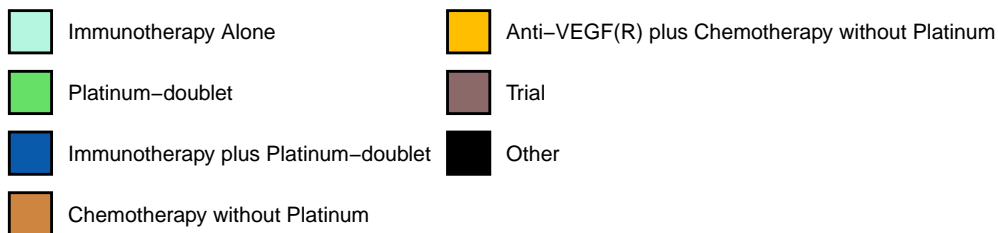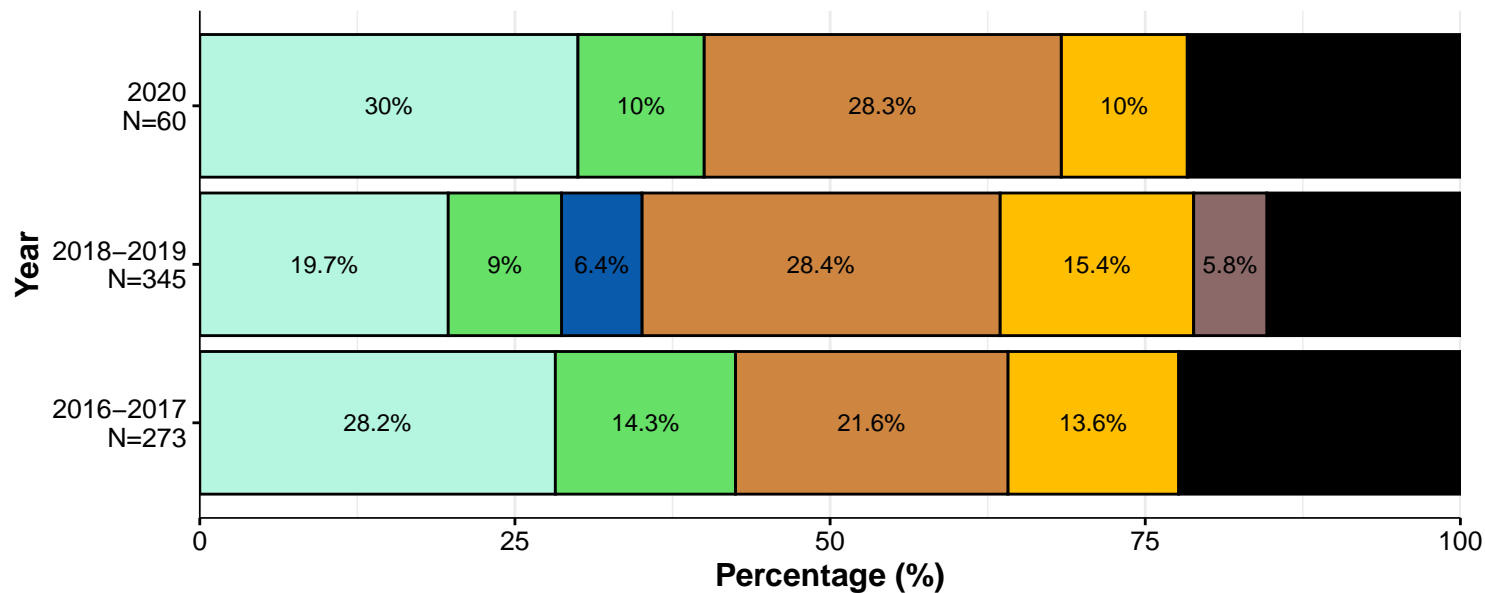

Supplement: Supplementary file 1 — Figure S1: Sample Selection Criteria Flow Chart. Figures S2a:,b. Second and Third‐Line Treatment Patterns for aNSCLC with Driver Alterations. Figures S2a:1–b.1 depict second‐line treatment patterns, and panels 2a.2–b.2 depict third‐line treatment patterns. All subfigures display treatments that exhibit a prevalence of ≥ 5% within each biomarker across the years as grouped in the bar charts. Otherwise, treatments demonstrating < 5% are regrouped into category called “Other.” Year represents the year when patients received first‐line treatment. Figure S3a:–c. Second and Third‐Line Treatment Patterns for aNSCLC without Driver Alterations. Figures S3a:1–c.1 depict second‐line treatment patterns, and panels 3a.2–c.2 depict third‐line treatment patterns. All subfigures display treatments that exhibit a prevalence of ≥ 5% within each biomarker across the years as grouped in the bar charts. Otherwise, treatments demonstrating < 5% are regrouped into category called “Other.” Year represents the year when patients received first‐line treatment. [file CAM4-15-e71736-s001.zip › cam471736-sup-0007-FigureS1-S49@Supplemental Figure 3b.2 PDL1 1to49.pdf]

# Treatment

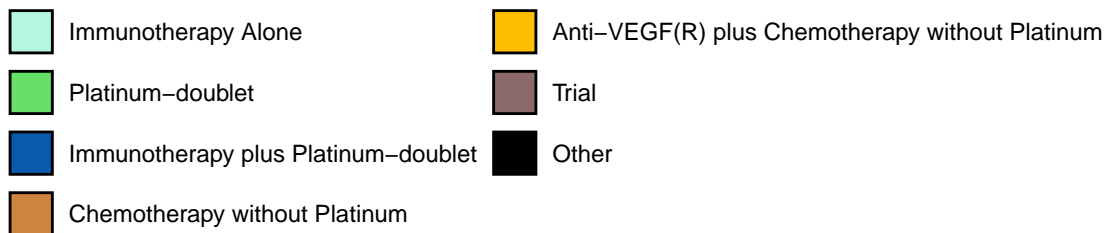

Year

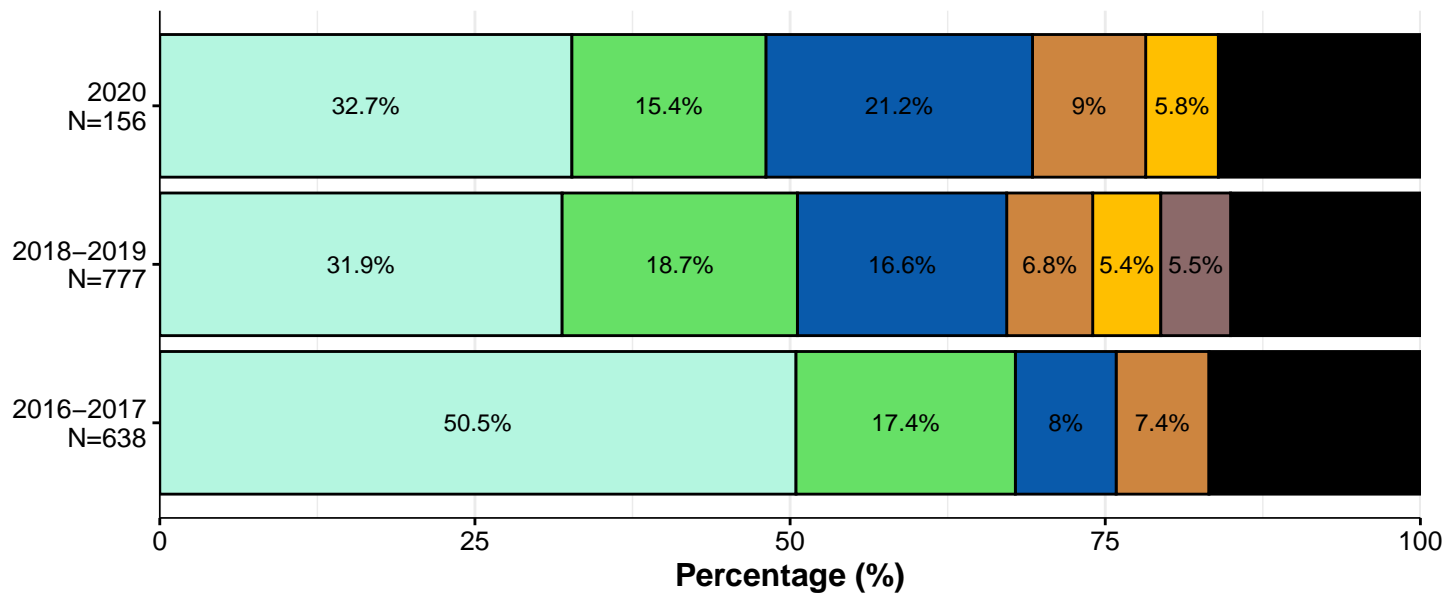

Supplement: Supplementary file 1 — Figure S1: Sample Selection Criteria Flow Chart. Figures S2a:,b. Second and Third‐Line Treatment Patterns for aNSCLC with Driver Alterations. Figures S2a:1–b.1 depict second‐line treatment patterns, and panels 2a.2–b.2 depict third‐line treatment patterns. All subfigures display treatments that exhibit a prevalence of ≥ 5% within each biomarker across the years as grouped in the bar charts. Otherwise, treatments demonstrating < 5% are regrouped into category called “Other.” Year represents the year when patients received first‐line treatment. Figure S3a:–c. Second and Third‐Line Treatment Patterns for aNSCLC without Driver Alterations. Figures S3a:1–c.1 depict second‐line treatment patterns, and panels 3a.2–c.2 depict third‐line treatment patterns. All subfigures display treatments that exhibit a prevalence of ≥ 5% within each biomarker across the years as grouped in the bar charts. Otherwise, treatments demonstrating < 5% are regrouped into category called “Other.” Year represents the year when patients received first‐line treatment. [file CAM4-15-e71736-s001.zip › cam471736-sup-0008-FigureS1-S50@Supplemental Figure 3c.1 PDL1 Gre50.pdf]

# Treatment

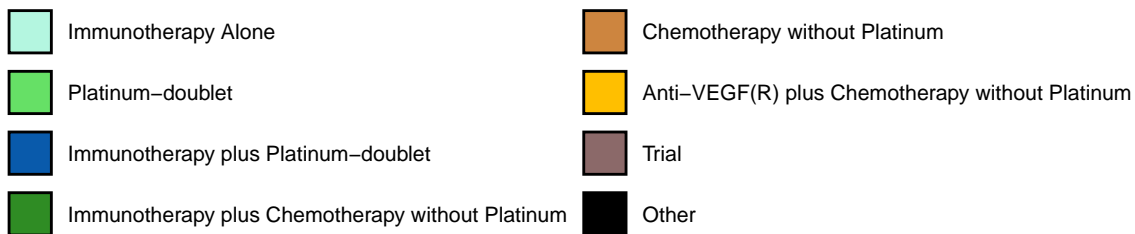

Year

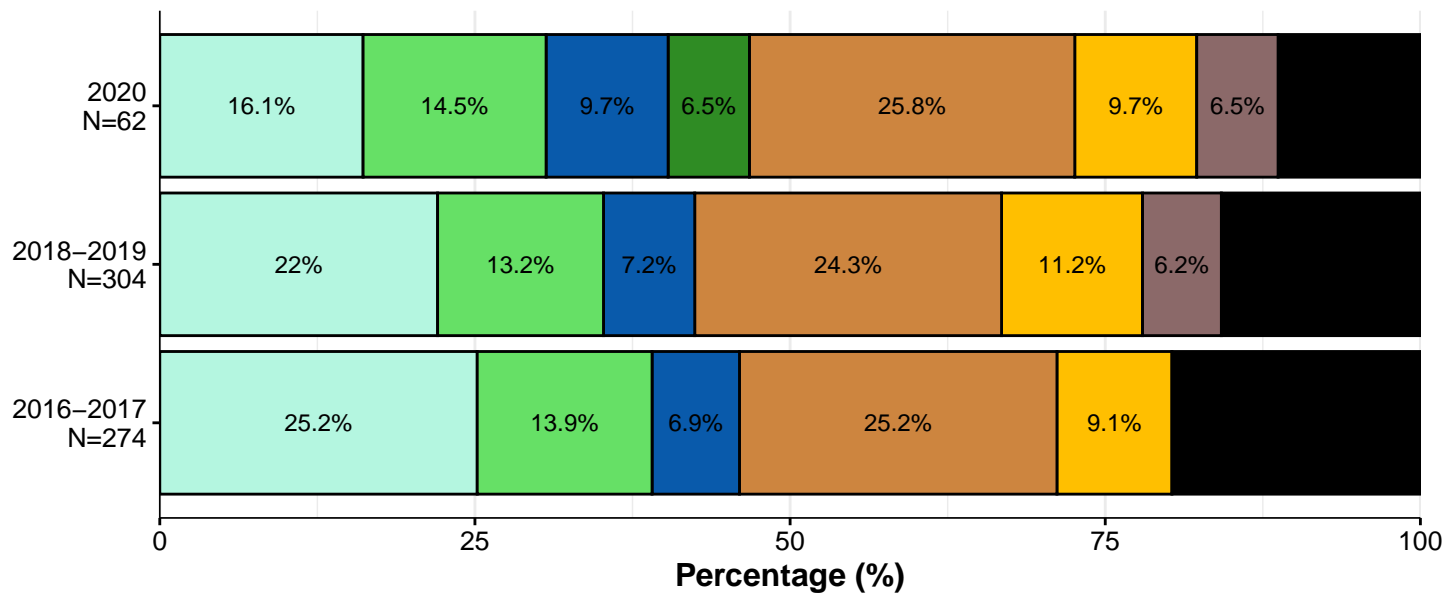

Supplement: Supplementary file 1 — Figure S1: Sample Selection Criteria Flow Chart. Figures S2a:,b. Second and Third‐Line Treatment Patterns for aNSCLC with Driver Alterations. Figures S2a:1–b.1 depict second‐line treatment patterns, and panels 2a.2–b.2 depict third‐line treatment patterns. All subfigures display treatments that exhibit a prevalence of ≥ 5% within each biomarker across the years as grouped in the bar charts. Otherwise, treatments demonstrating < 5% are regrouped into category called “Other.” Year represents the year when patients received first‐line treatment. Figure S3a:–c. Second and Third‐Line Treatment Patterns for aNSCLC without Driver Alterations. Figures S3a:1–c.1 depict second‐line treatment patterns, and panels 3a.2–c.2 depict third‐line treatment patterns. All subfigures display treatments that exhibit a prevalence of ≥ 5% within each biomarker across the years as grouped in the bar charts. Otherwise, treatments demonstrating < 5% are regrouped into category called “Other.” Year represents the year when patients received first‐line treatment. [file CAM4-15-e71736-s001.zip › cam471736-sup-0009-FigureS1-S50@Supplemental Figure 3c.2 PDL1 Gre50.pdf]

# Treatment

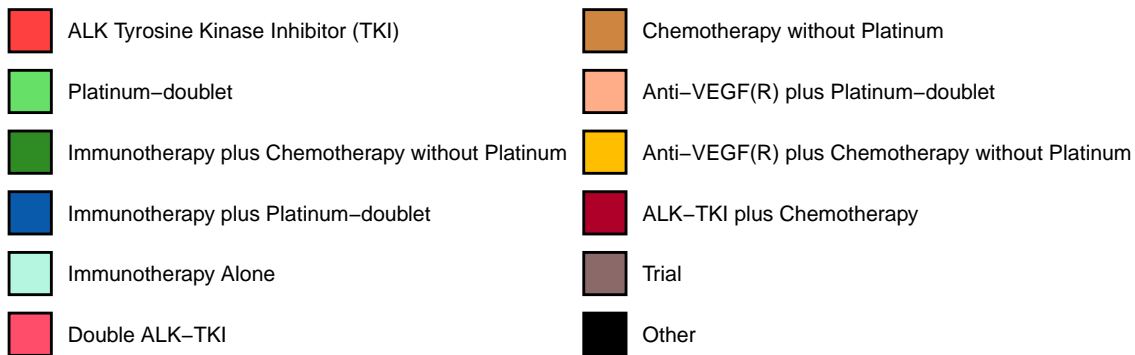

## Year

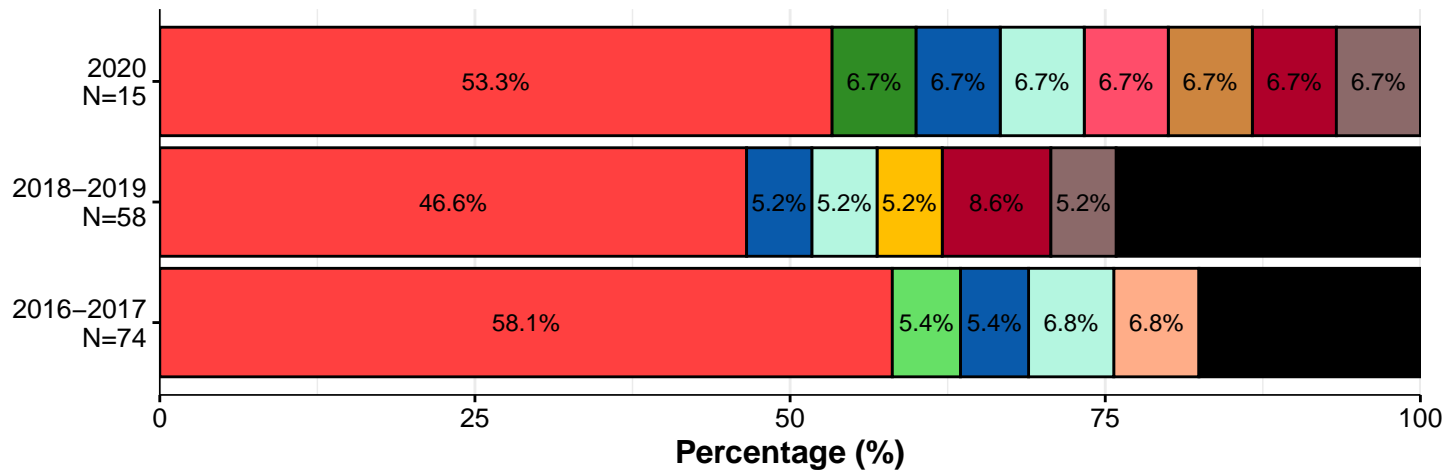

Supplement: Supplementary file 1 — Figure S1: Sample Selection Criteria Flow Chart. Figures S2a:,b. Second and Third‐Line Treatment Patterns for aNSCLC with Driver Alterations. Figures S2a:1–b.1 depict second‐line treatment patterns, and panels 2a.2–b.2 depict third‐line treatment patterns. All subfigures display treatments that exhibit a prevalence of ≥ 5% within each biomarker across the years as grouped in the bar charts. Otherwise, treatments demonstrating < 5% are regrouped into category called “Other.” Year represents the year when patients received first‐line treatment. Figure S3a:–c. Second and Third‐Line Treatment Patterns for aNSCLC without Driver Alterations. Figures S3a:1–c.1 depict second‐line treatment patterns, and panels 3a.2–c.2 depict third‐line treatment patterns. All subfigures display treatments that exhibit a prevalence of ≥ 5% within each biomarker across the years as grouped in the bar charts. Otherwise, treatments demonstrating < 5% are regrouped into category called “Other.” Year represents the year when patients received first‐line treatment. [file CAM4-15-e71736-s001.zip › cam471736-sup-0010-FigureS2-S2@Supplemental Figure 2a.2 ALK.pdf]

# Treatment

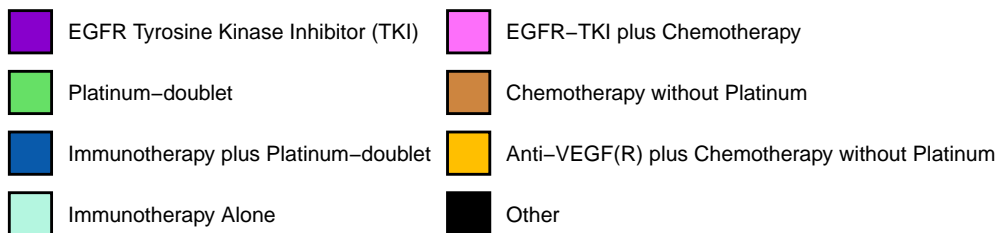

Year

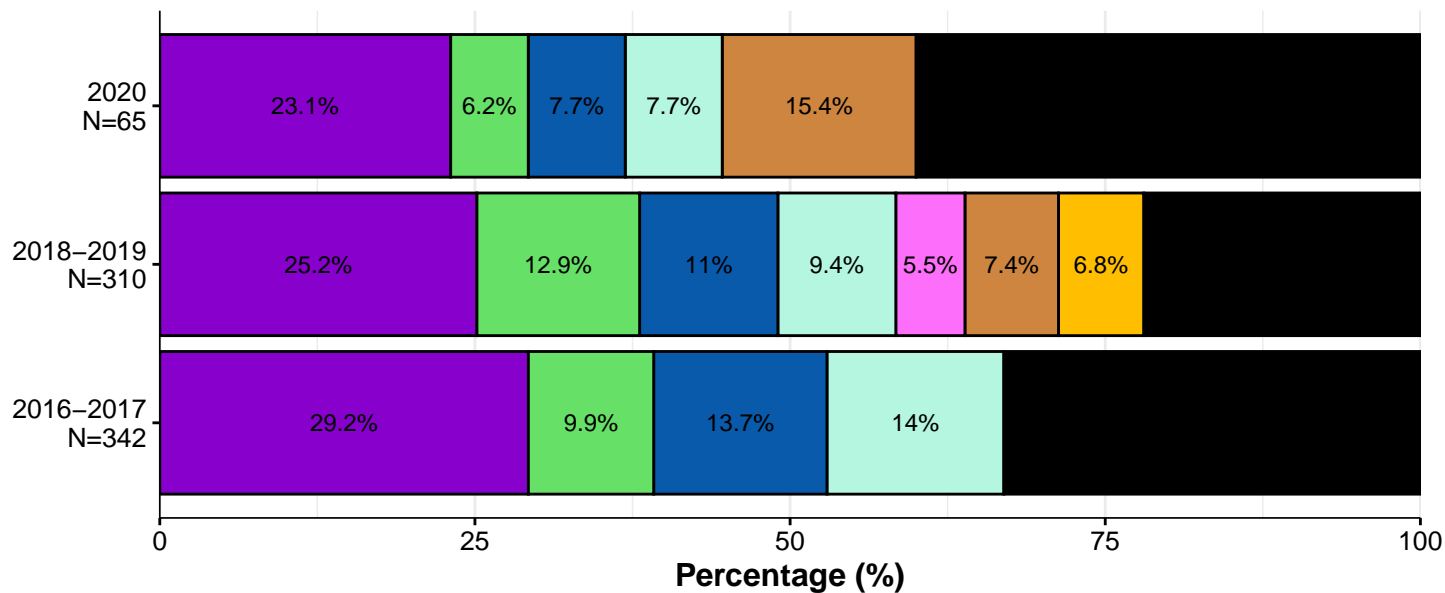

Supplement: Supplementary file 1 — Figure S1: Sample Selection Criteria Flow Chart. Figures S2a:,b. Second and Third‐Line Treatment Patterns for aNSCLC with Driver Alterations. Figures S2a:1–b.1 depict second‐line treatment patterns, and panels 2a.2–b.2 depict third‐line treatment patterns. All subfigures display treatments that exhibit a prevalence of ≥ 5% within each biomarker across the years as grouped in the bar charts. Otherwise, treatments demonstrating < 5% are regrouped into category called “Other.” Year represents the year when patients received first‐line treatment. Figure S3a:–c. Second and Third‐Line Treatment Patterns for aNSCLC without Driver Alterations. Figures S3a:1–c.1 depict second‐line treatment patterns, and panels 3a.2–c.2 depict third‐line treatment patterns. All subfigures display treatments that exhibit a prevalence of ≥ 5% within each biomarker across the years as grouped in the bar charts. Otherwise, treatments demonstrating < 5% are regrouped into category called “Other.” Year represents the year when patients received first‐line treatment. [file CAM4-15-e71736-s001.zip › cam471736-sup-0011-FigureS2-S2@Supplemental Figure 2b.2 EGFR.pdf]
