# Supplementary material for: Treatment Patterns Across Lines of Therapy for Advanced Non‐Small Cell Lung Cancer in the United States
Source: Cancer Med. 2026 Apr 20;15(4):e71736. doi: 10.1002/cam4.71736 (PMC13094514; doi:10.1002/cam4.71736)
Supplement: Supplementary file 2 — Table S1: Categorization of Regimens Based on Clinician Input, Therapeutic Targets, and Clinical Contexts Beyond NCCN Guidelines. [file CAM4-15-e71736-s002.docx]

**Supplemental Table 1: Categorization of Regimens Based on Clinician Input, Therapeutic Targets, and Clinical Contexts Beyond NCCN Guidelines**

|  | **LineGroup** ^a^ | **Line name (aNSCLC only)** |
| --- | --- | --- |
| 1 | ALK-TKI | Alectinib |
|  |  | Brigatinib |
|  |  | Ceritinib |
|  |  | Crizotinib |
|  |  | Lorlatinib |
| 2 | Anti-VEGF(R) and Chemotherapy without Platinum | Bevacizumab, Vinorelbine |
|  |  | Docetaxel, Ramucirumab |
|  |  | Bevacizumab, Paclitaxel Protein-Bound |
|  |  | Bevacizumab, Docetaxel |
|  |  | Bevacizumab, Docetaxel, Gemcitabine |
|  |  | Bevacizumab, Docetaxel, Ramucirumab |
|  |  | Bevacizumab, Gemcitabine |
|  |  | Bevacizumab, Gemcitabine, Paclitaxel Protein-Bound |
|  |  | Bevacizumab, Gemcitabine, Pemetrexed |
|  |  | Bevacizumab, Gemcitabine, Vinorelbine |
|  |  | Bevacizumab, Pemetrexed |
|  |  | Bevacizumab, Paclitaxel |
|  |  | Bevacizumab, Docetaxel, Paclitaxel Protein-Bound |
|  |  | Docetaxel, Gemcitabine, Ramucirumab |
|  |  | Docetaxel, Paclitaxel Protein-Bound, Ramucirumab |
|  |  | Gemcitabine, Ramucirumab |
|  |  | Gemcitabine, Ramucirumab, Vinorelbine |
|  |  | Irinotecan, Ramucirumab |
|  |  | Paclitaxel, Ramucirumab |
|  |  | Paclitaxel Protein-Bound, Ramucirumab |
| 3 | Anti-VEGF(R) and Platinum-doublet | Bevacizumab, Carboplatin, Pemetrexed |
|  |  | Bevacizumab, Carboplatin, Docetaxel |
|  |  | Bevacizumab, Carboplatin, Docetaxel, Paclitaxel |
|  |  | Bevacizumab, Carboplatin, Docetaxel, Paclitaxel Protein-Bound |
|  |  | Bevacizumab, Carboplatin, Gemcitabine |
|  |  | Bevacizumab, Carboplatin, Gemcitabine, Paclitaxel |
|  |  | Bevacizumab, Carboplatin, Gemcitabine, Pemetrexed |
|  |  | Bevacizumab, Carboplatin, Paclitaxel |
|  |  | Bevacizumab, Carboplatin, Paclitaxel, Pemetrexed |
|  |  | Bevacizumab, Carboplatin, Paclitaxel Protein-Bound |
|  |  | Bevacizumab, Cisplatin, Gemcitabine |
|  |  | Bevacizumab, Cisplatin, Paclitaxel |
|  |  | Bevacizumab, Cisplatin, Paclitaxel, Pemetrexed |
|  |  | Bevacizumab, Cisplatin, Pemetrexed |
|  |  | Bevacizumab, Carboplatin, Docetaxel, Gemcitabine |
|  |  | Bevacizumab, Carboplatin, Docetaxel, Paclitaxel, Ramucirumab |
|  |  | Bevacizumab, Carboplatin, Vinorelbine |
|  |  | Carboplatin, Etoposide, Ramucirumab |
|  |  | Carboplatin, Paclitaxel, Ramucirumab |
|  |  | Carboplatin, Paclitaxel Protein-Bound, Ramucirumab |
| 4 | Chemotherapy without Platinum | Docetaxel |
|  |  | Gemcitabine |
|  |  | Pemetrexed |
|  |  | Vinorelbine |
|  |  | Paclitaxel |
|  |  | Docetaxel, Gemcitabine |
|  |  | Docetaxel, Gemcitabine, Vinorelbine |
|  |  | Docetaxel, Paclitaxel |
|  |  | Docetaxel, Paclitaxel Protein-Bound |
|  |  | Docetaxel, Pemetrexed |
|  |  | Docetaxel, Vinorelbine |
|  |  | Etoposide |
|  |  | Gemcitabine, Irinotecan |
|  |  | Gemcitabine, Vinorelbine |
|  |  | Gemcitabine, Paclitaxel |
|  |  | Gemcitabine, Paclitaxel Protein-Bound |
|  |  | Irinotecan |
|  |  | Paclitaxel Protein-Bound |
|  |  | Paclitaxel Protein-Bound, Vinorelbine |
|  |  | Topotecan |
| 5 | EGFR-TKI | Osimertinib |
|  |  | Afatinib |
|  |  | Erlotinib |
|  |  | Dacomitinib |
|  |  | Gefitinib |
| 6 | EGFR-TKI and Chemotherapy | Afatinib, Carboplatin, Paclitaxel |
|  |  | Afatinib, Carboplatin, Pemetrexed |
|  |  | Afatinib, Paclitaxel |
|  |  | Afatinib, Paclitaxel Protein-Bound |
|  |  | Carboplatin, Osimertinib, Paclitaxel |
|  |  | Carboplatin, Erlotinib, Gemcitabine |
|  |  | Carboplatin, Erlotinib, Paclitaxel |
|  |  | Carboplatin, Erlotinib, Paclitaxel Protein-Bound |
|  |  | Carboplatin, Erlotinib, Pemetrexed |
|  |  | Carboplatin, Etoposide, Osimertinib |
|  |  | Carboplatin, Gefitinib, Pemetrexed |
|  |  | Carboplatin, Gemcitabine, Osimertinib |
|  |  | Carboplatin, Irinotecan, Osimertinib |
|  |  | Carboplatin, Osimertinib, Paclitaxel Protein-Bound |
|  |  | Carboplatin, Osimertinib, Pemetrexed |
|  |  | Cisplatin, Gemcitabine, Osimertinib |
|  |  | Docetaxel, Osimertinib |
|  |  | Erlotinib, Pemetrexed |
|  |  | Gemcitabine, Osimertinib |
|  |  | Lurbinectedin, Osimertinib |
|  |  | Osimertinib, Pemetrexed |
|  |  | Osimertinib, Paclitaxel |
|  |  | Osimertinib, Paclitaxel Protein-Bound |
|  |  | Osimertinib, Topotecan |
| 7 | Immunotherapy Alone | Pembrolizumab |
|  |  | Ipilimumab, Nivolumab |
|  |  | Nivolumab |
|  |  | Durvalumab |
|  |  | Atezolizumab |
|  |  | Cemiplimab |
|  |  | Cemiplimab, Durvalumab |
|  |  | Durvalumab, Pembrolizumab |
|  |  | Durvalumab, Tremelimumab-Actl |
|  |  | Ipilimumab, Pembrolizumab |
|  |  | Nivolumab, Pembrolizumab |
| 8 | Immunotherapy, Anti-VEGF(R), and Platinum-doublet | Atezolizumab, Bevacizumab, Carboplatin, Paclitaxel |
|  |  | Atezolizumab, Bevacizumab, Carboplatin, Paclitaxel Protein-Bound |
|  |  | Atezolizumab, Bevacizumab, Carboplatin, Paclitaxel Protein-Bound, Pembrolizumab, Pemetrexed |
|  |  | Atezolizumab, Bevacizumab, Carboplatin, Pemetrexed |
|  |  | Atezolizumab, Bevacizumab, Carboplatin, Docetaxel, Paclitaxel |
|  |  | Atezolizumab, Bevacizumab, Cisplatin, Paclitaxel Protein-Bound |
|  |  | Bevacizumab, Carboplatin, Docetaxel, Pembrolizumab, Pemetrexed |
|  |  | Bevacizumab, Carboplatin, Nivolumab, Pemetrexed |
|  |  | Bevacizumab, Carboplatin, Paclitaxel, Pembrolizumab, Pemetrexed |
|  |  | Bevacizumab, Carboplatin, Ipilimumab, Nivolumab, Paclitaxel, Pemetrexed |
|  |  | Bevacizumab, Carboplatin, Paclitaxel Protein-Bound, Pembrolizumab |
|  |  | Bevacizumab, Carboplatin, Pembrolizumab, Pemetrexed |
|  |  | Carboplatin, Docetaxel, Pembrolizumab, Pemetrexed, Ramucirumab |
| 9 | Immunotherapy and Chemotherapy without Platinum | Pembrolizumab, Pemetrexed |
|  |  | Atezolizumab, Docetaxel |
|  |  | Atezolizumab, Etoposide |
|  |  | Atezolizumab, Gemcitabine |
|  |  | Atezolizumab, Paclitaxel |
|  |  | Atezolizumab, Paclitaxel Protein-Bound |
|  |  | Atezolizumab, Pemetrexed |
|  |  | Cemiplimab, Pemetrexed |
|  |  | Docetaxel, Pembrolizumab |
|  |  | Docetaxel, Gemcitabine, Nivolumab |
|  |  | Docetaxel, Gemcitabine, Pembrolizumab |
|  |  | Docetaxel, Irinotecan, Pembrolizumab |
|  |  | Docetaxel, Nivolumab |
|  |  | Docetaxel, Pembrolizumab, Pemetrexed |
|  |  | Durvalumab, Pemetrexed |
|  |  | Etoposide, Pembrolizumab |
|  |  | Gemcitabine, Ipilimumab, Nivolumab |
|  |  | Gemcitabine, Nivolumab |
|  |  | Gemcitabine, Nivolumab, Vinorelbine |
|  |  | Gemcitabine, Paclitaxel Protein-Bound, Pembrolizumab |
|  |  | Gemcitabine, Pembrolizumab |
|  |  | Gemcitabine, Pembrolizumab, Vinorelbine |
|  |  | Ipilimumab, Nivolumab, Pemetrexed |
|  |  | Ipilimumab, Nivolumab, Vinorelbine |
|  |  | Nivolumab, Paclitaxel |
|  |  | Nivolumab, Paclitaxel Protein-Bound |
|  |  | Nivolumab, Pemetrexed |
|  |  | Nivolumab, Vinorelbine |
|  |  | Paclitaxel, Pembrolizumab |
|  |  | Paclitaxel Protein-Bound, Pembrolizumab |
|  |  | Pembrolizumab, Pemetrexed, Vinorelbine |
|  |  | Pembrolizumab, Vinorelbine |
| 10 | Immunotherapy and Platinum-doublet | Carboplatin, Pembrolizumab, Pemetrexed |
|  |  | Atezolizumab, Carboplatin, Etoposide |
|  |  | Atezolizumab, Carboplatin, Pemetrexed |
|  |  | Atezolizumab, Carboplatin, Paclitaxel |
|  |  | Atezolizumab, Carboplatin, Paclitaxel, Pembrolizumab |
|  |  | Atezolizumab, Carboplatin, Paclitaxel Protein-Bound |
|  |  | Atezolizumab, Carboplatin, Paclitaxel Protein-Bound, Pembrolizumab |
|  |  | Atezolizumab, Cisplatin, Paclitaxel |
|  |  | Carboplatin, Nivolumab, Paclitaxel Protein-Bound |
|  |  | Carboplatin, Cemiplimab, Paclitaxel |
|  |  | Carboplatin, Cemiplimab, Pemetrexed |
|  |  | Carboplatin, Docetaxel, Paclitaxel, Pembrolizumab |
|  |  | Carboplatin, Docetaxel, Pembrolizumab |
|  |  | Carboplatin, Docetaxel, Pembrolizumab, Pemetrexed |
|  |  | Carboplatin, Durvalumab, Etoposide |
|  |  | Carboplatin, Durvalumab, Gemcitabine |
|  |  | Carboplatin, Durvalumab, Paclitaxel |
|  |  | Carboplatin, Durvalumab, Paclitaxel, Pembrolizumab |
|  |  | Carboplatin, Durvalumab, Paclitaxel Protein-Bound |
|  |  | Carboplatin, Durvalumab, Paclitaxel Protein-Bound, Tremelimumab-Actl |
|  |  | Carboplatin, Durvalumab, Pembrolizumab, Pemetrexed |
|  |  | Carboplatin, Durvalumab, Pemetrexed |
|  |  | Carboplatin, Etoposide, Nivolumab |
|  |  | Carboplatin, Etoposide, Pembrolizumab |
|  |  | Carboplatin, Etoposide, Pembrolizumab, Pemetrexed |
|  |  | Carboplatin, Gemcitabine, Ipilimumab, Nivolumab |
|  |  | Carboplatin, Gemcitabine, Necitumumab, Pembrolizumab |
|  |  | Carboplatin, Gemcitabine, Paclitaxel, Pembrolizumab |
|  |  | Carboplatin, Gemcitabine, Paclitaxel Protein-Bound, Pembrolizumab |
|  |  | Carboplatin, Gemcitabine, Pembrolizumab |
|  |  | Carboplatin, Gemcitabine, Pembrolizumab, Pemetrexed |
|  |  | Carboplatin, Ipilimumab, Nivolumab, Paclitaxel |
|  |  | Carboplatin, Ipilimumab, Nivolumab, Paclitaxel Protein-Bound |
|  |  | Carboplatin, Ipilimumab, Nivolumab, Pemetrexed |
|  |  | Carboplatin, Nivolumab, Paclitaxel |
|  |  | Carboplatin, Nivolumab, Paclitaxel Protein-Bound |
|  |  | Carboplatin, Nivolumab, Paclitaxel Protein-Bound, Pembrolizumab |
|  |  | Carboplatin, Nivolumab, Pembrolizumab, Pemetrexed |
|  |  | Carboplatin, Nivolumab, Pemetrexed |
|  |  | Carboplatin, Paclitaxel, Pembrolizumab |
|  |  | Carboplatin, Paclitaxel, Pembrolizumab, Pemetrexed |
|  |  | Carboplatin, Paclitaxel Protein-Bound, Pembrolizumab |
|  |  | Carboplatin, Paclitaxel Protein-Bound, Pembrolizumab, Pemetrexed |
|  |  | Cisplatin, Docetaxel, Durvalumab |
|  |  | Cisplatin, Gemcitabine, Pembrolizumab |
|  |  | Cisplatin, Ipilimumab, Nivolumab, Pemetrexed |
|  |  | Cisplatin, Irinotecan, Pembrolizumab |
|  |  | Cisplatin, Paclitaxel, Pembrolizumab |
|  |  | Cisplatin, Paclitaxel Protein-Bound, Pembrolizumab |
|  |  | Cisplatin, Pembrolizumab, Pemetrexed |
| 11 | KRAS G12C-Inhibitor | Adagrasib |
|  |  | Adagrasib, Sotorasib |
|  |  | Sotorasib |
| 12 | Non-NSCLC Treatment | Abemaciclib |
|  |  | Abiraterone |
|  |  | Leuprolide |
|  |  | Alpelisib |
|  |  | Imatinib |
|  |  | Anastrozole |
|  |  | Apalutamide |
|  |  | Avelumab |
|  |  | Axitinib |
|  |  | Azacitidine |
|  |  | Ivosidenib |
|  |  | Venetoclax |
|  |  | BcgVaccine |
|  |  | Bendamustine, Obinutuzumab |
|  |  | Capecitabine |
|  |  | Fluorouracil, Irinotecan, Leucovorin |
|  |  | Oxaliplatin |
|  |  | Hydroxyurea |
|  |  | Rituximab |
|  |  | Tamoxifen |
|  |  | Bicalutamide |
|  |  | Binimetinib, Encorafenib |
|  |  | Bleomycin |
|  |  | Bortezomib, Daratumumab |
|  |  | Lenalidomide |
|  |  | Bosutinib |
|  |  | Cabazitaxel |
|  |  | Pemigatinib |
|  |  | Cabozantinib |
|  |  | Cetuximab |
|  |  | Cladribine |
|  |  | Copanlisib |
|  |  | Cyclophosphamide |
|  |  | Doxorubicin |
|  |  | Vincristine |
|  |  | Rituximab-Arrx |
|  |  | Fludarabine, Mesna |
|  |  | Rituximab/Hyaluronidase |
|  |  | Cytarabine |
|  |  | Darolutamide, Triptorelin |
|  |  | Dasatinib |
|  |  | Decitabine |
|  |  | Degarelix |
|  |  | Pertuzumab, Trastuzumab |
|  |  | Ifosfamide |
|  |  | DoxorubicinPegylatedLiposomal |
|  |  | Elacestrant, Letrozole |
|  |  | EnasidenibMesylate |
|  |  | EnfortumabVedotin-Ejfv |
|  |  | Enzalutamide |
|  |  | Erdafitinib |
|  |  | Eribulin |
|  |  | Everolimus |
|  |  | Exemestane |
|  |  | Irinotecan Liposomal |
|  |  | Levoleucovorin Panitumumab |
|  |  | Mitomycin |
|  |  | Fulvestrant |
|  |  | Palbociclib |
|  |  | Ibrutinib |
|  |  | Thalidomide |
|  |  | Lenvatinib |
|  |  | Methotrexate |
|  |  | Midostaurin |
|  |  | Neratinib |
|  |  | Nilotinib |
|  |  | Nilutamide |
|  |  | Niraparib, Olaparib |
|  |  | Ofatumumab |
|  |  | Pazopanib |
|  |  | Porfimer |
|  |  | Relugolix |
|  |  | Rucaparib |
|  |  | Ruxolitinib |
|  |  | Sorafenib |
|  |  | Temozolomide |
|  |  | Temsirolimus |
|  |  | Trastuzumab-Anns |
|  |  | Tretinoin |
|  |  | Vorinostat |
| 13 | Platinum-doublet | Carboplatin, Paclitaxel |
|  |  | Carboplatin, Pemetrexed |
|  |  | Cisplatin, Gemcitabine, Necitumumab |
|  |  | Carboplatin, Paclitaxel Protein-Bound |
|  |  | Carboplatin, Docetaxel |
|  |  | Carboplatin, Docetaxel, Paclitaxel |
|  |  | Carboplatin, Docetaxel, Pemetrexed |
|  |  | Carboplatin, Gemcitabine |
|  |  | Carboplatin, Etoposide |
|  |  | Carboplatin, Etoposide, Paclitaxel |
|  |  | Carboplatin, Etoposide, Paclitaxel, Pemetrexed |
|  |  | Carboplatin, Etoposide, Paclitaxel Protein-Bound |
|  |  | Carboplatin, Etoposide, Pemetrexed |
|  |  | Carboplatin, Gemcitabine, Necitumumab |
|  |  | Carboplatin, Gemcitabine, Paclitaxel |
|  |  | Carboplatin, Gemcitabine, Paclitaxel Protein-Bound |
|  |  | Carboplatin, Gemcitabine, Pemetrexed |
|  |  | Carboplatin, Gemcitabine, Vinorelbine |
|  |  | Carboplatin, Irinotecan |
|  |  | Carboplatin, Paclitaxel, Pemetrexed |
|  |  | Carboplatin, Paclitaxel, Vinorelbine |
|  |  | Carboplatin, Paclitaxel Protein-Bound |
|  |  | Carboplatin, Paclitaxel Protein-Bound, Pemetrexed |
|  |  | Carboplatin, Vinorelbine |
|  |  | Cisplatin, Docetaxel |
|  |  | Cisplatin, Docetaxel, Etoposide |
|  |  | Cisplatin, Docetaxel, Pemetrexed |
|  |  | Cisplatin, Etoposide |
|  |  | Cisplatin, Etoposide, Gemcitabine |
|  |  | Cisplatin, Etoposide, Paclitaxel |
|  |  | Cisplatin, Etoposide, Pemetrexed |
|  |  | Cisplatin, Gemcitabine |
|  |  | Cisplatin, Gemcitabine, Necitumumab |
|  |  | Cisplatin, Gemcitabine, Paclitaxel |
|  |  | Cisplatin, Pemetrexed |
|  |  | Cisplatin, Paclitaxel |
|  |  | Cisplatin, Paclitaxel Protein-Bound |
|  |  | Cisplatin, Vinorelbine |
| 14 | Single-agent Platinum | Cisplatin |
|  |  | Carboplatin |
| 15 | Trial | Any line of treatment that includes Clinical Study Drug |
| 16 | Other ^b^ | Ado-TrastuzumabEmtansine |
|  |  | Ado-TrastuzumabEmtansine, Atezolizumab |
|  |  | Ado-TrastuzumabEmtansine, Atezolizumab, Carboplatin, Paclitaxel |
|  |  | Ado-TrastuzumabEmtansine, Carboplatin, Gemcitabine |
|  |  | Ado-TrastuzumabEmtansine, Fam-TrastuzumabDeruxtecan-Nxki |
|  |  | Ado-TrastuzumabEmtansine, Nivolumab |
|  |  | Ado-TrastuzumabEmtansine, Ramucirumab |
|  |  | Afatinib, Amivantamab-Vmjw |
|  |  | Afatinib, Atezolizumab |
|  |  | Afatinib, Atezolizumab, Bevacizumab-Bvzr, Ramucirumab |
|  |  | Afatinib, Bevacizumab |
|  |  | Afatinib, Bevacizumab, Carboplatin, Paclitaxel |
|  |  | Afatinib, Bevacizumab, Carboplatin, Pemetrexed |
|  |  | Afatinib, Carboplatin, Osimertinib, Pembrolizumab, Pemetrexed |
|  |  | Afatinib, Carboplatin, Pembrolizumab, Pemetrexed |
|  |  | Afatinib, Docetaxel, Ramucirumab |
|  |  | Afatinib, Erlotinib |
|  |  | Afatinib, Gefitinib |
|  |  | Afatinib, Nivolumab |
|  |  | Afatinib, Osimertinib |
|  |  | Afatinib, Pembrolizumab |
|  |  | Afatinib, Pembrolizumab, Pemetrexed |
|  |  | Afatinib, Ramucirumab |
|  |  | Alectinib, Trastuzumab |
|  |  | Alectinib, Atezolizumab |
|  |  | Alectinib, Bevacizumab |
|  |  | Alectinib, Bevacizumab, Brigatinib |
|  |  | Alectinib, Bevacizumab, Carboplatin, Pemetrexed |
|  |  | Alectinib, Bevacizumab, Pemetrexed |
|  |  | Alectinib, Pembrolizumab |
|  |  | Alectinib, Brigatinib |
|  |  | Alectinib, Carboplatin, Etoposide |
|  |  | Alectinib, Carboplatin, Gemcitabine |
|  |  | Alectinib, Carboplatin, Lorlatinib, Pemetrexed |
|  |  | Alectinib, Carboplatin, Paclitaxel |
|  |  | Alectinib, Carboplatin, Paclitaxel, Pembrolizumab |
|  |  | Alectinib, Carboplatin, Pembrolizumab, Pemetrexed |
|  |  | Alectinib, Carboplatin, Pemetrexed |
|  |  | Alectinib, Cisplatin, Pemetrexed |
|  |  | Alectinib, Crizotinib |
|  |  | Alectinib, Lorlatinib |
|  |  | Alectinib, Nivolumab |
|  |  | Alectinib, Osimertinib |
|  |  | Alectinib, Pembrolizumab |
|  |  | Amivantamab-Vmjw |
|  |  | Amivantamab-Vmjw, Osimertinib |
|  |  | Atezolizumab, Bevacizumab |
|  |  | Atezolizumab, Bevacizumab, Carboplatin, Osimertinib, Paclitaxel |
|  |  | Atezolizumab, Bevacizumab, Osimertinib |
|  |  | Atezolizumab, Bevacizumab, Paclitaxel |
|  |  | Atezolizumab, Bevacizumab, Paclitaxel, Pembrolizumab, Pemetrexed |
|  |  | Atezolizumab, Bevacizumab, Paclitaxel Protein-Bound |
|  |  | Atezolizumab, Bevacizumab, Osimertinib, Paclitaxel Protein-Bound |
|  |  | Atezolizumab, Bevacizumab Osimertinib, Pemetrexed |
|  |  | Atezolizumab, Capmatinib, Carboplatin, Etoposide |
|  |  | Atezolizumab, Carboplatin, Erlotinib, Etoposide |
|  |  | Atezolizumab, Carboplatin, Etoposide, Osimertinib |
|  |  | Atezolizumab, Carboplatin, Osimertinib, Paclitaxel Protein-Bound |
|  |  | Atezolizumab, Osimertinib |
|  |  | Bevacizumab, Carboplatin |
|  |  | Bevacizumab, Carboplatin, Erlotinib, Pemetrexed |
|  |  | Bevacizumab, Carboplatin, Lorlatinib, Paclitaxel |
|  |  | Bevacizumab, Carboplatin, Osimertinib, Paclitaxel |
|  |  | Bevacizumab, Carboplatin, Osimertinib, Paclitaxel Protein-Bound |
|  |  | Bevacizumab, Carboplatin, Osimertinib, Pemetrexed |
|  |  | Bevacizumab, Crizotinib |
|  |  | Bevacizumab, Erlotinib |
|  |  | Bevacizumab, Erlotinib, Osimertinib |
|  |  | Bevacizumab, Gefitinib |
|  |  | Bevacizumab, Nivolumab |
|  |  | Bevacizumab, Nivolumab, Pemetrexed |
|  |  | Bevacizumab, Osimertinib |
|  |  | Bevacizumab, Osimertinib, Pemetrexed |
|  |  | Bevacizumab, Pembrolizumab |
|  |  | Bevacizumab, Pembrolizumab, Pemetrexed |
|  |  | Bevacizumab, Pembrolizumab, Vinorelbine |
|  |  | Bevacizumab, Pemetrexed, Trastuzumab |
|  |  | Bevacizumab, Cabozantinib, Osimertinib |
|  |  | Bevacizumab, Carboplatin, Dabrafenib, Osimertinib, Pemetrexed, Trametinib |
|  |  | Bevacizumab-, Carboplatin, Dabrafenib, Pemetrexed, Trametinib |
|  |  | Bevacizumab, Carboplatin, Etoposide, Lorlatinib |
|  |  | Bevacizumab, Carboplatin, Lorlatinib, Pemetrexed |
|  |  | Bevacizumab, Carboplatin, Nivolumab |
|  |  | Bevacizumab, Carboplatin, Pemetrexed, Sotorasib |
|  |  | Bevacizumab, Ipilimumab, Nivolumab |
|  |  | Bevacizumab, Lorlatinib |
|  |  | Bevacizumab, Cisplatin, Lorlatinib, Pemetrexed |
|  |  | Bevacizumab, Irinotecan, Osimertinib |
|  |  | Bevacizumab, Sotorasib |
|  |  | Brigatinib, Carboplatin, Pembrolizumab, Pemetrexed |
|  |  | Brigatinib, Carboplatin, Pemetrexed |
|  |  | Brigatinib, Crizotinib |
|  |  | Brigatinib, Lorlatinib |
|  |  | Brigatinib, Paclitaxel |
|  |  | Brigatinib, Pembrolizumab, Pemetrexed |
|  |  | Brigatinib, Pemetrexed |
|  |  | Cabozantinib, Osimertinib |
|  |  | Capmatinib |
|  |  | Capmatinib, Carboplatin, Osimertinib, Pemetrexed |
|  |  | Capmatinib, Crizotinib |
|  |  | Capmatinib, Gemcitabine |
|  |  | Capmatinib, Lorlatinib |
|  |  | Capmatinib, Osimertinib |
|  |  | Carboplatin, Crizotinib, Paclitaxel |
|  |  | Carboplatin, Crizotinib, Pembrolizumab, Pemetrexed |
|  |  | Carboplatin, Crizotinib, Pemetrexed |
|  |  | Osimertinib, Paclitaxel, Trastuzumab |
|  |  | Carboplatin, Entrectinib, Pembrolizumab, Pemetrexed |
|  |  | Carboplatin, Erlotinib, Osimertinib, Pemetrexed |
|  |  | Carboplatin, Erlotinib, Pembrolizumab, Pemetrexed |
|  |  | Carboplatin, Etoposide, Osimertinib, Pembrolizumab, Pemetrexed |
|  |  | Carboplatin, Lorlatinib, Paclitaxel Protein-Bound |
|  |  | Carboplatin, Lorlatinib, Pembrolizumab, Pemetrexed |
|  |  | Carboplatin, Lorlatinib, Pemetrexed |
|  |  | Carboplatin, Nivolumab |
|  |  | Carboplatin, Osimertinib, Paclitaxel Protein-Bound, Pembrolizumab |
|  |  | Carboplatin, Osimertinib, Pembrolizumab, Pemetrexed |
|  |  | Carboplatin, Pembrolizumab |
|  |  | Carboplatin, Pembrolizumab, Pemetrexed, Trastuzumab |
|  |  | Carboplatin, Pemetrexed, Sotorasib |
|  |  | Carboplatin, Pemetrexed, Trastuzumab |
|  |  | Ceritinib, Gemcitabine |
|  |  | Cisplatin, Durvalumab, Etoposide, Lorlatinib |
|  |  | Cisplatin, Pembrolizumab |
|  |  | Cobimetinib |
|  |  | Crizotinib, Erlotinib |
|  |  | Crizotinib, Lorlatinib |
|  |  | Crizotinib, Osimertinib |
|  |  | Crizotinib, Pembrolizumab |
|  |  | Crizotinib, Pembrolizumab, Pemetrexed |
|  |  | Crizotinib, Trastuzumab |
|  |  | Dabrafenib, Trametinib |
|  |  | Dabrafenib, Osimertinib |
|  |  | Dabrafenib, Osimertinib, Trametinib |
|  |  | Dacomitinib, Osimertinib |
|  |  | Docetaxel, Durvalumab, Pembrolizumab, Ramucirumab |
|  |  | Docetaxel, Erlotinib, Ramucirumab |
|  |  | Docetaxel, Nivolumab, Ramucirumab |
|  |  | Docetaxel, Fam-TrastuzumabDeruxtecan-Nxki, Ramucirumab |
|  |  | Docetaxel, Lorlatinib |
|  |  | Docetaxel, Lorlatinib, Ramucirumab |
|  |  | Docetaxel, Osimertinib, Ramucirumab |
|  |  | Docetaxel, Paclitaxel Protein-Bound, Pembrolizumab, Ramucirumab |
|  |  | Docetaxel, Pembrolizumab, Ramucirumab |
|  |  | Docetaxel, Ramucirumab, Sotorasib |
|  |  | Durvalumab, Gefitinib |
|  |  | Durvalumab, Osimertinib |
|  |  | Entrectinib |
|  |  | Entrectinib, Lorlatinib |
|  |  | Erlotinib, Nivolumab |
|  |  | Erlotinib, Osimertinib |
|  |  | Erlotinib, Pembrolizumab |
|  |  | Erlotinib, Pembrolizumab, Pemetrexed |
|  |  | Erlotinib, Ramucirumab |
|  |  | Fam-TrastuzumabDeruxtecan-Nxki |
|  |  | Pembrolizumab, Ramucirumab |
|  |  | Gefitinib, Osimertinib |
|  |  | Gefitinib, Pembrolizumab |
|  |  | Gemcitabine, Lorlatinib |
|  |  | Gemcitabine, Osimertinib, Trastuzumab |
|  |  | Ipilimumab, Nivolumab, Sotorasib |
|  |  | Osimertinib, Ramucirumab |
|  |  | Larotrectinib |
|  |  | Larotrectinib, Lorlatinib |
|  |  | Osimertinib, Trastuzumab |
|  |  | Lorlatinib, Nivolumab, Pemetrexed |
|  |  | Lorlatinib, Pemetrexed |
|  |  | Mobocertinib |
|  |  | Nivolumab, Osimertinib |
|  |  | Osimertinib, Pembrolizumab |
|  |  | Osimertinib, Pembrolizumab, Pemetrexed |
|  |  | Osimertinib, Pralsetinib |
|  |  | Osimertinib, Ramucirumab |
|  |  | Osimertinib, Trametinib |
|  |  | Osimertinib, Vemurafenib |
|  |  | Pembrolizumab, Ramucirumab |
|  |  | Pembrolizumab, Ramucirumab, Sotorasib |
|  |  | Pembrolizumab, Ramucirumab, Vinorelbine |
|  |  | Pembrolizumab, Sotorasib |
|  |  | Pralsetinib |
|  |  | Ramucirumab |
|  |  | Selpercatinib |
|  |  | Tepotinib |
|  |  | Trametinib |
|  |  | Vemurafenib |

^a^ All Line Groups across all biomarker groups with ≥5% prevalence across lines of therapy.

^b^ The “Other” category consolidates LineGroups with <5% prevalence across various biomarker groups into a single group.
