# Supplementary material for: Treatment Patterns Across Lines of Therapy for Advanced Non‐Small Cell Lung Cancer in the United States
Source: Cancer Med. 2026 Apr 20;15(4):e71736. doi: 10.1002/cam4.71736 (PMC13094514; doi:10.1002/cam4.71736)
Supplement: Supplementary file 3 — Table S2: Adjusted Association of Biomarker Group with Treatment Overall Discontinuation at One Year Post‐First Line Therapy. [file CAM4-15-e71736-s003.docx]

**Supplementary Table 2. Adjusted Association of Biomarker Group with Treatment Overall Discontinuation at One Year Post-First Line Therapy^1^**

|  | **Odds Ratio (95% Confidence Interval), P-Value** |
| --- | --- |
| **Biomarker Group**  (ref = EGFR) | <0.001^2^ |
| ALK | 0.63 (0.47, 0.82), 0.001 |
| PD-L1 <1% | 2.69 (2.39, 3.03), <0.001 |
| PD-L1 1-49% | 2.37 (2.11, 2.68), <0.001 |
| PD-L1 ≥50% | 2.01 (1.79, 2.26), <0.001 |
| **Age, years^3^** | 1.02 (1.01, 1.02), <0.001 |
| **Race/Ethnicity**  (ref = White) | <0.001^4^ |
| Asian | 0.68 (0.53, 0.86) , 0.001 |
| Black or African American | 0.91 (0.80, 1.04), 0.18 |
| Other | 1.17 (1.03, 1.33), 0.02 |
| Unknown | 1.16 (1.06, 1.27), 0.001 |
| **Sex**  (ref = Female) | 1.41 (1.31, 1.51), <0.001 |
| **ECOG Status**  (ref = 0-1) | <0.001^5^ |
| 2-4 | 1.35 (1.25, 1.46), <0.001 |
| Missing | 1.91 (1.71, 2.14), <0.001 |

1 Overall discontinuation was defined as death within one year without receiving second-line therapy; all other patients were considered non-discontinued.

2 Overall P-Value to indicate the association between biomarker group and overall discontinuation at one year post-first line therapy

3 Patients with a birth year of [data cut-off year - 85] or earlier may have an adjusted birth year in Flatiron Health datasets due to patient deidentification requirements.

4 Overall P-Value to indicate the association between race/ethnicity and overall discontinuation at one year post-first line therapy

5 Overall P-Value to indicate the association between ECOG status and overall discontinuation at one year post-first line therapy
